# Climate action literacy interventions increase commitments to more effective mitigation behaviors

**DOI:** 10.1093/pnasnexus/pgaf191

**Published:** 2025-06-09

**Authors:** Danielle Goldwert, Yash Patel, Kristian Steensen Nielsen, Matthew H Goldberg, Madalina Vlasceanu

**Affiliations:** Department of Psychology, New York University, New York, NY 10003, USA; Department of Environmental Social Sciences, Stanford University, Stanford, CA 94305, USA; Department of Management, Society and Communication, Copenhagen Business School, Frederiksberg 2000, Denmark; Yale Program on Climate Change Communication, Yale University, New Haven, CT 06511, USA; Department of Psychology, New York University, New York, NY 10003, USA; Department of Environmental Social Sciences, Stanford University, Stanford, CA 94305, USA

**Keywords:** intervention, climate, behavior, misperception, spillover

## Abstract

Reducing lifestyle carbon emissions is a critical component of decarbonizing society. However, people hold substantial misperceptions about the relative efficacy of different behavioral changes, such as comprehensively recycling or avoiding long flights, and these misperceptions may lead to the suboptimal allocation of resources. In a preregistered experiment in the United States, we tested the effects of two literacy interventions on correcting misperceptions and increasing commitments toward more effective individual-level climate actions. Participants (*n* = 3,895) were randomly assigned to one of three experimental conditions: a Prediction condition, in which they were asked to rank the relative mitigation potential of 21 climate behaviors after which they received feedback; an Information condition, in which they were passively exposed to information about the relative mitigation potential of the same behaviors; and a no-information Control condition. Both the Prediction and Information interventions led to more accurate efficacy perceptions and increased commitments to engage in higher-impact individual-level actions relative to the Control group. Greater initial misperceptions were associated with larger shifts in commitments, such that participants reduced commitments to behaviors that were overestimated and increased commitments to behaviors that were underestimated in their carbon reduction potential. However, we also found evidence for a negative spillover effect from individual to collective actions: participants in the literacy conditions decreased their commitments to collective climate actions such as voting or marching, suggesting an unintended consequence of interventions focusing solely on individual-level actions.

Significance Statement Many people misunderstand which personal actions meaningfully reduce carbon emissions, potentially misdirecting efforts. By experimentally providing information about the relative mitigation potential of each behavior, we show that people can recalibrate their understanding and increase their commitment to more impactful individual-level climate behaviors, such as avoiding long flights or eating less meat. However, interventions focusing exclusively on individual-level behaviors run the risk of reducing willingness to engage in collective climate actions, such as voting or attending climate marches. These findings suggest that climate literacy interventions can help individuals optimize their personal lifestyle choices in ways that align with climate mitigation efforts. Still, they caution intervention designers against focusing solely on individual-level actions, as they can reduce engagement with collective action.

## Introduction

Human-driven climate change poses one of the most urgent challenges of our time, requiring rapid reductions in greenhouse gas (GHG) emissions to mitigate its worst impacts (1). While international agreements and large-scale policies are critical pathways to climate mitigation, individuals' decisions (e.g. how often people fly, if they eat red meat, what type of car they drive) also play a substantial role in achieving net zero emissions (2, 3), in some calculations accounting for at least 40% of emissions reductions needed by 2050 (1). However, despite increasing concern about climate change around the world (4), individuals are not engaging in behaviors commensurate with the degree of the threat (5). This conceptual–behavioral discrepancy could be explained by a knowledge deficit model (6, 7), by which people may not know which climate actions to engage in to substantially impact climate change. For example, people might think recycling is among the most effective climate actions (8) despite being orders of magnitude less impactful than other behaviors such as transportation decisions (2, 9). Indeed, studies suggest that people hold large misperceptions regarding the effectiveness of household, transportation, or recycling behaviors (10, 11). Given the additional constraint of limited bandwidth or limited resources to engage in a multitude of climate-relevant behaviors (12), misperceptions about the efficacy of each behavior can lead people to engage in actions with limited effect on carbon emissions, resulting in suboptimal deployment of effort and resources (13). Here, we assess the potential of climate action literacy interventions to correct misperceptions and nudge commitments to activities with higher mitigation potential.

Increasing literacy through informational interventions has had mixed effects on the various facets of climate change mitigation. While educating the public about climate change has been found to increase belief in anthropogenic climate change (14), education interventions often have had limited effectiveness in promoting climate mitigation behaviors (15). Recent work has explored the potential of behavioral feedback. For instance, people who tracked their personal carbon footprint through smartphone apps significantly reduced their emissions by adjusting their habits (16). In another study, people who received feedback on the carbon footprint of their grocery product choices decreased high-emissions purchases (e.g. beef) and lowered their overall emissions (17). Such interventions may have calibrated people's understanding of the relative emissions associated with various behaviors and choices, nudging them toward engaging in more effective climate actions. Here, we empirically test this mechanism, investigating whether correcting misperceptions about the mitigation potential of various climate actions leads to corresponding behavioral commitments.

The cognitive processes triggered during information exposure may also play a role in the effectiveness of literacy interventions in inducing sustained behavioral commitments. For instance, information communicated through passive exposure may not be integrated into one's belief system and corresponding behavioral signatures (18–20). On the contrary, mechanisms leading to successfully updating mental models have been shown to rely on active processing of new information, for example, through prediction errors (21). Generating predictions about one's environment is a ubiquitous process of the cognitive system, and when these predictions are invalidated, they can lead to substantial conceptual (22) and behavioral changes (23, 24). For instance, triggering large prediction errors in a prediction-then-feedback paradigm led to more evidence incorporation and belief updating compared with passive evidence exposure, an effect that was consistent across the political–ideological spectrum (21). Here, we build on this work and test whether a passive climate action literacy intervention or a prediction-then-feedback intervention is more effective at correcting climate action misperceptions and increasing commitments to higher climate mitigation potential actions.

In addition to the effectiveness of climate actions in reducing climate change, another critical determinant of behavioral uptake is plasticity (25–28). We use the term behavioral plasticity to refer to the degree to which a behavior can be changed, given the actual or perceived financial, time, cognitive, and social costs that shape ease of adoption. For example, installing solar panels may involve high financial costs, whereas switching to public transportation could require significant time or logistical planning, and shifting to a vegetarian diet may present social or cultural barriers. Studies suggest that people are often more likely to engage in lower-cost climate actions (4) and actions perceived as having higher climate mitigation efficacy (29). We therefore aim to identify the actions that meet both of these criteria (i.e. low cost, high efficacy), representing a strategic starting point for policy makers seeking to realize climate-relevant behavior changes.

Beyond individual-level climate actions, engaging in collective action represents an equally critical means to address the growing climate crisis (30–33). Within this context, while some have argued that focusing on individual-level behaviors might reduce the likelihood of people engaging in collective action through a negative spillover process (34), others have proposed that, instead, engaging in one type of climate action has the potential to unlock willingness to engage in additional types of action through a positive spillover process (35–37). Moreover, it is important to note that individual-level actions can produce indirect or systemic impacts beyond their direct carbon reductions. For instance, consumer demand shifts can influence corporate decision-making, spur reputational concerns, or foster market-driven transitions toward sustainable products and services. Here, we also investigate whether increasing commitments to individual-level climate actions have a positive or negative spillover effect on collective actions, such as voting or participating in climate demonstrations.

Finally, we investigate the longitudinal effects of these literacy interventions. Most studies to date have documented immediate intervention effects (38), with a burgeoning body of work suggesting rapid rates of decay of such interventions (39, 40). Accordingly, we assessed the effects of interest after a 1-week delay to gauge their persistence. The durability of effects is particularly important from an implementation perspective, as policy makers are more likely to consider interventions that have the potential for longer-term impact.

To investigate, we conducted a large-scale preregistered experiment on a sample of 3,895 US residents. We first measured their baseline commitments (pretest) to a set of 21 individual (e.g. eating less meat) and 5 collective (e.g. voting) climate mitigation behaviors (Table 1). We then randomly assigned them to one of three conditions: Prediction, Information, or Control. Participants in the Prediction condition (*n* = 1,297) were instructed to predict the relative ranking, in terms of carbon mitigation potential, of each of the 21 individual-level behaviors; after making each prediction, participants were given feedback and shown the actual relative effectiveness of each behavior. Participants in the Information condition (*n* = 1,303) were directly exposed to information about the relative carbon mitigation potential of each of the 21 individual-level behaviors. Participants in the control condition (*n* = 1,295) engaged in an unrelated task. We then measured participants' commitments to each of the behaviors again (at posttest and after 1 week) and the perceived effectiveness of each behavior at reducing carbon emissions. Finally, we measured the perceived behavioral plasticity of each behavior and assessed participants' demographic characteristics (see Materials and Methods for additional information).

**Table 1.** List of the 26 behaviors used in this study.

*Individual-level behaviors* “Can you commit to this action?”
1. Use more efficient appliances (e.g. change your lightbulbs)
2. Comprehensively recycle for at least 1 year
3. Use less energy related to clothing (e.g. hang dry clothing and wash clothes in cold water) for at least 1 year
4. Eat 30% more vegetarian food (e.g. be vegetarian for one additional meal per day) for at least 1 year
5. Produce renewable electricity (e.g. install small-scale residential solar photovoltaic)
6. Car-pool/share (e.g. become a member of a car-club, reduce the number of cars in your household, or ride-share with at least two persons in a car) for at least 1 year
7. Install smart metering (i.e. measure how much gas and electricity you’re using via a remote connection to your energy supplier)
8. Reduce avoidable food waste for at least 1 year
9. Eat 60% more vegetarian food (e.g. be vegetarian for two additional meals per day) for at least 1 year
10. Take less transport by air (e.g. avoid medium flights, or shift from airplane to renewable train) for at least 1 year
11. Increase energy efficiency (e.g. buy a more efficient car)
12. Shift from fossil fuel public transport to renewable public transport (e.g. shift from fossil fuel bus/train to renewable train)
13. Adopt a vegan diet for at least 1 year
14. Adopt a vegetarian diet (e.g. go from omnivore to vegetarian) for at least 1 year
15. Shift from fuel-powered car to a renewable electric car for at least 1 year
16. Shift to lower-carbon meats (e.g. shift one-third of the beef calories to either pork or poultry) for at least 1 year
17. Shift from fossil fuel car to renewable public transport (e.g. shift from fossil car to renewable train)
18. Shift to active transport (e.g. bike and ebike instead of taking the car) for at least 1 year
19. Use renewable electricity (e.g. buy green energy) for at least 1 year
20. Take one less transatlantic flight for at least 1 year
21. Not purchase/adopt a dog
*System-level behaviors*
1. Vote for proclimate candidates
2. Attend a climate march/demonstration
3. Change your financial institution (if it invests in fossil fuels)
4. Donate to an environmental nonprofit
5. Promote climate action at work

The individual-level behaviors are ordered from least (item 1) to most (item 21) effective at reducing carbon emissions.

## Results

### Descriptives

#### What are high-efficacy high-plasticity individual-level climate behaviors?

**Fig. 1.**
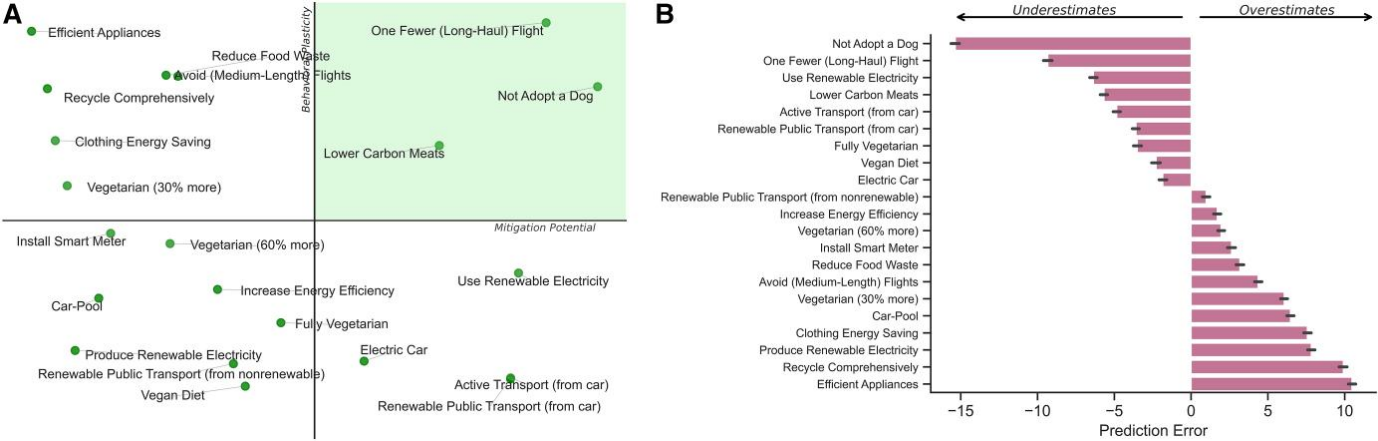
A) Map of individual-level climate behaviors on two key dimensions: carbon mitigation potential as detected in a meta-analysis by Ivanova et al. (2) (*x*-axis) and behavioral plasticity as assessed here (*y*-axis). B) Prediction errors (i.e. the difference between the predicted and actual rank) associated with each of the 21 individual-level climate-relevant behaviors assessed. Error bars represent 95% CIs.

First, we mapped the space of individual-level climate behaviors measured here on the two key dimensions of interest: carbon mitigation potential (*x*-axis) and behavioral plasticity (*y*-axis). We found that taking one fewer flight, not adopting a dog, or eating lower-carbon meats were the three behaviors with the highest levels of carbon emissions reductions and behavioral plasticity (Fig. 1A).

#### Do people misperceive the effectiveness of individual-level climate behaviors?

Next, we calculated the prediction errors made by participants in the Prediction condition, representing the difference between their predicted rank and the actual rank of each of the 21 behaviors on the carbon emissions scale. For example, if a participant selected the answer 10 (on a scale from 1 to 21), and the correct answer was 4, the prediction error for that behavior was 6, meaning that they overestimated the relative effectiveness of that behavior at reducing carbon emissions. Conversely, if a participant selected the answer 2 on that same question, their prediction error was −2, meaning that they underestimated that behavior's relative effectiveness. We found that participants significantly underestimated the mitigation potential of behaviors like not adopting a dog or taking one fewer long-haul flights, while greatly overestimating the potential of behaviors like using efficient appliances or recycling comprehensively (Fig. 1B). This result held across demographic groups (political ideology, age, income, education, and gender; see Table S1; see also Figs. S1–S5 for prediction error patterns by subgroup).

Hypothesis 1: Literacy interventions will increase accuracy about climate behavior efficacy relative to the control group.

**Fig. 2.**
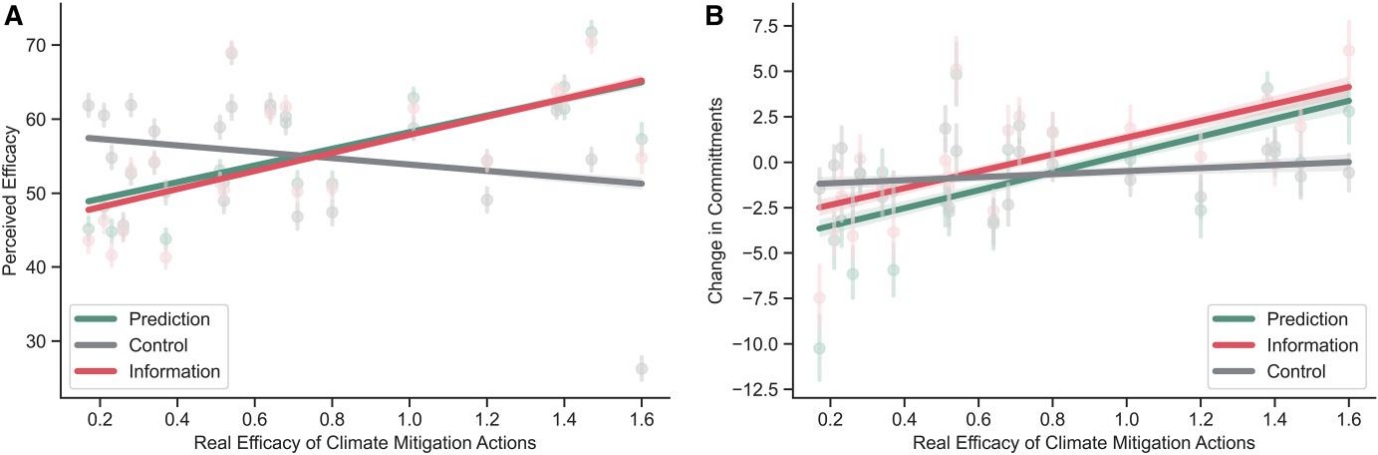
A) Real vs. perceived climate action efficacy. Steeper positive slopes correspond to more accurate predictions of action efficacy. *X*-axis units are absolute carbon mitigation potential per year for each behavior. B) Real climate action efficacy as a function of change in commitments from pretest to posttest, in each of the three conditions.

We first tested whether participants in the Experimental conditions would have more accurate perceptions of individual-level climate action effectiveness than participants in the Control condition. We conducted a linear mixed-effects model with perceived efficacy as the dependent variable, condition (Prediction, Information, Control), and actual efficacy as predictors, and by-participant random intercepts. We found significant main effects for both the Prediction (*b* = 15.56, SE = 0.40, *t* = 39.26, *P* < 0.001) and Information conditions (*b* = 16.52, SE = 0.40, *t* = 41.72, *P* < 0.001) relative to Control (Fig. 2A; Table S2). The effects remained significant at a 1-week follow-up, pointing to their durability (Table S15). These results support our first hypothesis and suggest that literacy interventions durably increase accuracy about climate behaviors' efficacy at reducing carbon emissions.

Contrary to our prediction, however, the Information condition was significantly more accurate than the Prediction condition (*b* = 0.96, SE = 0.40, *t* = 2.42, *P* = 0.015), suggesting that there is no added benefit to engaging in an active predictive process in this context and that passive information exposure is enough to correct misperceptions regarding climate action efficacy (Table S3). This effect was also significant at the 1-week follow-up (Table S16).

Hypothesis 2: Literacy interventions will increase commitments to engage in more effective climate behaviors relative to the control group.

We next examined whether improving the accuracy of perceived effectiveness leads to shifts in actual commitments toward actions with higher mitigation potential. We ran a linear mixed-effects model with change in commitment from pretest to posttest as the dependent variable, actual efficacy, condition, and their interactions as fixed effects including by-participant random intercepts. In line with hypothesis 2, we found that participants in the Prediction (*b* = 4.08, SE = 0.41, *t* = 10.05, *P* < 0.001) and Information conditions (*b* = 3.78, SE = 0.41, *t* = 9.32, *P* < 0.001) better aligned their commitments with the more consequential behaviors relative to the Control condition (Fig. 2B; Table S4). These results indicate that correcting misperceptions can lead to increases in commitment to more impactful behaviors. There was no significant difference in commitment changes between the Information and Prediction conditions (*b* = −0.30, SE = 0.41, *t* = −0.74, *P* = 0.460; Table S5), suggesting that both forms of corrective intervention can increase commitments to more impactful behaviors. However, these effects did not remain significant after a 1-week delay, pointing to their short-term influence (Tables S17 and 18).

We then conducted a causal mediation analysis to examine whether perceptions of efficacy potentially mediated the relationship between the intervention conditions (combined) and changes in behavior commitments. We found a significant average causal mediation effect (ACME), indicating that perceptions of efficacy mediated 11.08% of the total effect (TE) of condition on commitment (ACME = 0.0488, 95% CI [0.0094, 0.09], *P* = 0.012). The average direct effect (ADE) was also significant (ADE = 0.3916, 95% CI [0.0794, 0.70], *P* = 0.014), as was the TE (TE = 0.4404, 95% CI [0.1310, 0.74], *P* = 0.010), suggesting efficacy perception partially mediated the effects of the literacy interventions on climate action commitments (but see 41 for limitations of mediation analysis, such as its lack of ability to make causal inferences or distinguish between plausible alternative models; see Table S6). At follow-up, the mediation analysis showed the indirect effect was no longer significant, suggesting that the mediation effect was not durable (Table S19).

Hypothesis 3: Larger prediction errors will lead to increased changes in commitment to corresponding actions.

**Fig. 3.**
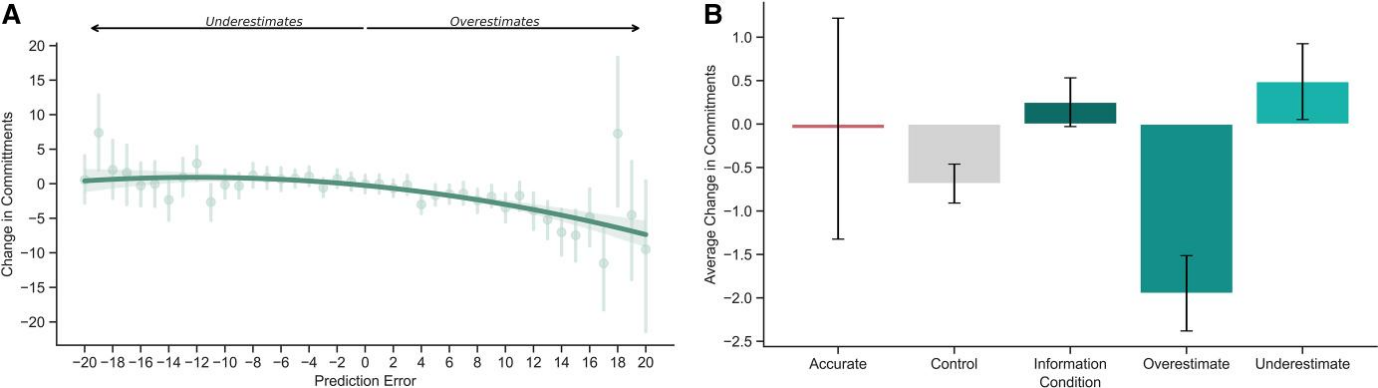
A) Prediction errors (difference between real and perceived relative effectiveness of climate actions) as a function of changes in commitments in corresponding actions, from pretest to posttest. Larger underestimates with correction increase commitment, while overestimates with correction reduce commitment. B) Experimental conditions (Control, Information, and Prediction split into Overestimates–Underestimates–Accurate) as a function of commitments to engage in climate actions.

Within the Prediction condition, we conducted a mixed-effects model with change in commitment as the dependent variable and prediction error as a quadratic predictor. This analysis yielded support for hypothesis 3, as both the linear term (*b* = −262.57, SE = 25.60, *t* = −10.26, *P* < 0.001) and the quadratic term (*b* = −115.78, SE = 25.78, *t* = −4.49, *P* < 0.001) for prediction error were significantly negative, indicating that as prediction errors increased, commitments to corresponding actions decreased. However, the lower AIC (Akaike Information Criterion) (195,198.8 vs. 195,239.7) in the quadratic model suggested a nonlinear relationship (Fig. 3A; Tables S7 and S8; see Table S20 for follow-up quadratic model; see also Table S21 for the linear version).

Further analyses of overestimated, underestimated, or accurately estimated actions shed additional light on the effects of prediction errors on commitments. We ran a linear mixed model with change in commitments as the dependent variable, group (Control, Information, Overestimated, Underestimated, or Accurate items) as the independent variable, including by-participant random intercepts. We found that, relative to the control condition, participants *decreased* their commitments to engage in behaviors they overestimated the effectiveness of (*b* = −1.34, SE = 0.34, *t* = −3.91, *P* < 0.001). Further, participants *increased* their commitments to engage in behaviors they underestimated the effectiveness of (*b* = 1.29, SE = 0.35, *t* = 3.72, *P* < 0.001; Fig. 3B), and this effect held after a 1-week delay (Table S22; see Table S9 for full model results by prediction error group).

### Political affiliation as a moderator

**Fig. 4.**
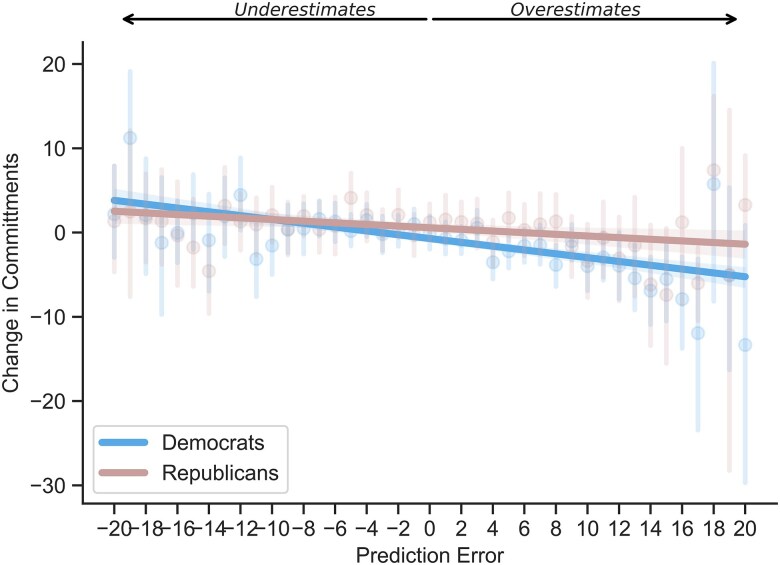
Prediction errors (difference between real and perceived relative effectiveness of climate actions) as a function of changes in commitments in corresponding actions, from pretest to posttest, split by political affiliation.

Given the stark political polarization of climate change, which is especially pronounced in the United States (42), we investigated potential ideological moderators of the effects of prediction errors on commitments. In a linear mixed-effects model, we found that, compared with Republicans, Democrats were more sensitive to making prediction errors about the effectiveness of climate actions, changing their behavioral commitments more according to the new information learned (interaction *b* = 0.14, SE = 0.05, *t* = 2.30, *P* = 0.003; Fig. 4; Table S10). However, the interaction between prediction error and political affiliation was not significant at follow-up (Table S23).

Hypothesis 4: Even though the actual effectiveness of the collective actions is not revealed, there will be a change in commitment to these actions in the experimental relative to the control group.

**Fig. 5.**
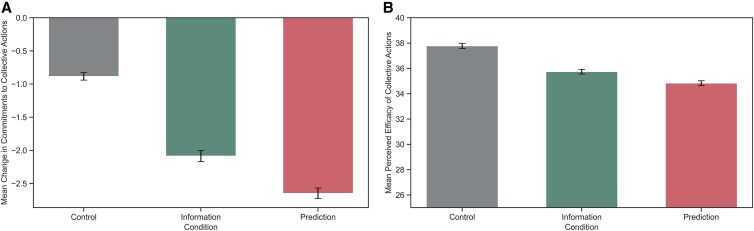
A) Changes in commitments from pretest to posttest in collective actions as a function of experimental condition. B) Changes in perceptions of efficacy of collective actions as a function of experimental condition. Error bars represent SEs of the mean.

We next examined whether changes in perceptions and commitments to individual-level actions would spillover to collective behaviors, about which participants received no direct effectiveness information. We found evidence supporting significant differences in commitments to collective actions between conditions (*F*[2, 51,489] = 147.49, *P* < 0.001; Fig. 5A; see Table S11 for pairwise comparison results). Post hoc comparisons using the Tukey HSD test indicated that participants in the Information condition (*M* = −2.09, SD = 0.08) and Prediction condition (*M* = −2.65, SD = 0.08) significantly *decreased* their commitments to engaging in collective action compared with the control condition (*M* = −0.88, SD = 0.06). This points to a negative spillover effect. The result held for efficacy perceptions: the Information condition (*M* = −2.09, SD = 10.91) and the Prediction condition (*M* = −2.64, SD = 10.47) significantly decreased efficacy perceptions of collective actions compared with the control condition (*M* = −0.88, SD = 7.39) (Fig. 5B). In a mediation analysis, we found that efficacy perception mediated the relationship between condition and commitments to collective actions, mediation supported by a significant indirect effect (ACME = 0.016, 95% CI [0.004, 0.03], *P* = 0.010; Table S12). However, this indirect effect was not significant at follow-up (Table S24).

### Exploratory analysis

#### What is a stronger predictor of pretest commitments, behavioral plasticity or perceived efficacy?

**Fig. 6.**
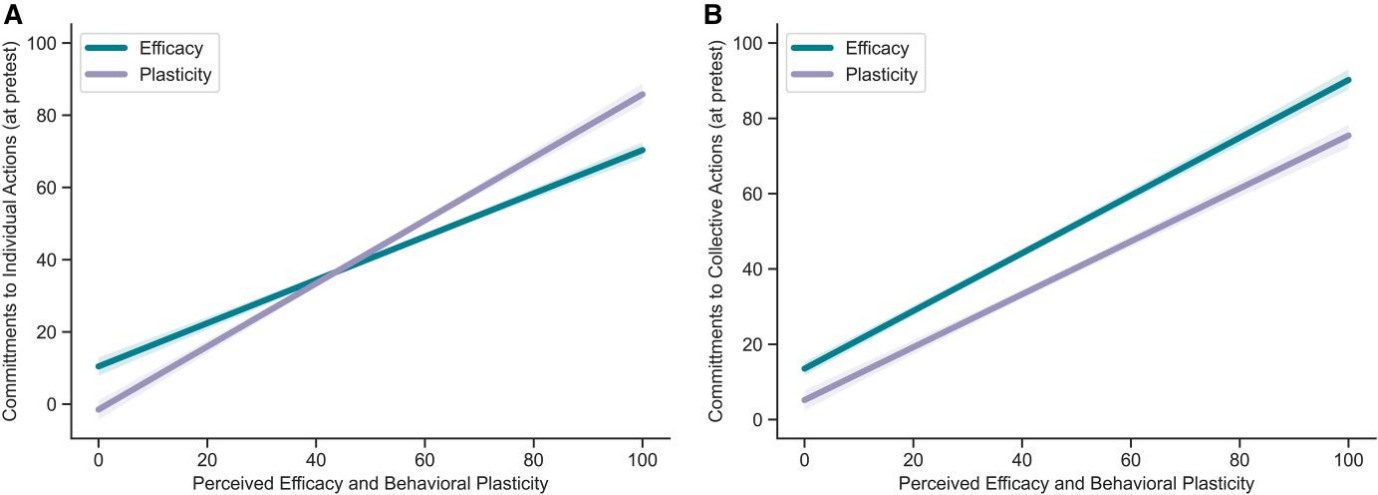
A) Pretest commitments to individual-level actions as a function of perceived behavioral plasticity and perceived efficacy. B) Pretest commitments to collective-level actions as a function of perceived behavioral plasticity and perceived efficacy.

To determine whether behavioral plasticity or perceived efficacy was a stronger predictor of behavioral commitments to individual actions, we ran a linear mixed-effects model using the data in the control condition. The model included commitments at pretest as the dependent variable, plasticity and perceived efficacy as fixed effects, with by-participants random effects. We found that plasticity is a stronger predictor of behavioral commitments (*b* = 0.65, SE = 0.005, *t* = 131.9, *P* < 0.001) than perceived mitigation potential (*b* = 0.148, SE = 0.007, *t* = 21.7, *P* < 0.001) (Fig 6A; Table S13), a conclusion supported by a significant *Z*-test for slope differences (*Z* = 71.21, *P* < 0.001). When running the model using commitments to collective actions instead, perceived efficacy (*b* = 0.57, SE = 0.01, *t* = 106.37, *P* < 0.001) was a stronger predictor than plasticity (*b* = 0.38, SE = .01, *t* = 68.91, *P* < 0.001) (Fig 6B; Table S14) supported by a significant *Z*-test for slope differences (*Z* = 21.02, *P* < 0.001). These results suggest that different motivational mechanisms predict engaging in individual-level vs. collective-level actions. While perceived ease of adoption is more important when considering engaging in a new lifestyle behavior, perceived efficacy is more important when considering engaging in collective action.

## Discussion

In a preregistered experiment in the United States (*n* = 3,946), we tested the effectiveness of two climate literacy interventions at increasing the accuracy of perceptions of the mitigation potential of individual-level climate actions, such as recycling, flying, or consuming meat. First, we found that participants initially held large misperceptions about the relative effectiveness of various climate actions in reducing carbon emissions. This finding aligns with prior work (10, 11) and the knowledge deficit model (6, 7). For instance, participants overestimated the carbon emissions reductions from using efficient appliances or recycling and underestimated those from avoiding one long flight.

We also found that correcting these misperceptions using information interventions durably increased accuracy, which in turn led to behavioral commitments better aligned with the behaviors' mitigation potential (i.e. higher commitments to more impactful behaviors such as flying less, lower commitments to less impactful actions such as recycling). This finding advances prior work on the effectiveness of educational interventions in increasing beliefs in climate change (14), suggesting that beyond beliefs, climate literacy interventions have the potential to shape behavioral commitments to act proenvironmentally.

We found no differences in behavioral commitments between the two literacy interventions tested, a direct information exposure intervention and a prediction-then-feedback intervention. This result counters research showing that large prediction errors lead to more belief updating than passive exposure to evidence (21), pointing to the boundary conditions of the prediction-then-feedback strategy. This finding also advances prior research on behavioral feedback aiming at aligning consumer decisions with sustainable choices in order to reduce emissions (16, 17), providing a mechanistic account behind the effects and suggesting that information alone may have achieved similar behavioral outcomes to the feedback programs. Indeed, the lack of differences in commitments between the two interventions tested here is promising for the direct information intervention strategy, given the scalability and lower cost of this approach.

Moreover, participants in the Prediction condition decreased their commitments to actions they overestimated the impact of (such as comprehensively recycling) and increased their commitments to actions they underestimated the impact of (such as taking one less flight per year), more strongly aligning their commitments to the efficacy of their behaviors. This effect was particularly pronounced among Democratic participants, who were more sensitive to the effects of prediction errors than Republican participants, in line with prior work on belief updating across political boundaries (21).

We also found evidence of negative spillover effects, whereby participants in the intervention conditions reported decreased willingness to engage in collective-level climate actions such as voting or attending climate marches. This finding suggests that even interventions aimed at increasing accuracy about the effectiveness of climate actions and commitments to higher-impact behaviors can have unintended consequences. Accordingly, we recommend that climate communicators, policy makers, and other practitioners not omit collective-level actions or civic engagement from climate literacy interventions and other educational programs. However, the degree of negative spillover may be context-dependent. For example, when government action is perceived as gridlocked or ineffectual, individuals might see limited value in collective political engagement—thus the choice may effectively be between individual action and no action at all. Future interventions might reduce negative spillover by emphasizing both individual-level behaviors and the efficacy of collective-level actions—whether aimed at government policy, corporate initiatives, or community-based movements.

Finally, in exploratory analyses, we found that different precursors of behavior are more important when deciding whether to engage in individual vs. collective climate actions. Behavioral plasticity (or perceived ease of adoption) is more important for individual-level actions, such as switching to public transportation or a vegetarian diet. However, perceived efficacy is more important for collective-level actions such as attending a climate march or promoting sustainability in one's workplace. This finding suggests that different intervention pathways might be more appropriate for individual vs. collective actions. For example, instead of focusing on their efficacy, reducing the barriers to adopting climate-friendly lifestyle behaviors might prove more effective at scale. While efficacy interventions might be effective in the short term, their long-term effects might prove limited since they are not the primary driver of lifestyle behavioral change, as documented here. For collective actions, emphasizing their efficacy might be the most effective pathway for intervention, although future work should empirically validate these assumptions.

It is important to note that the emission reductions estimates associated with the climate actions tested here can vary regionally and may become outdated as the electric grid continues to decarbonize and baseline behaviors shift (2). Consequently, we interpret these rankings primarily as a comparative tool to illustrate higher- vs. lower-impact actions rather than as static, universal estimates of absolute emissions. Future work could refine these rankings further by accounting for localized grid factors or recent technology adoption trends in different parts of the country.

It is also worth noting that our study did not provide participants with explicit information on the carbon mitigation efficacy of collective actions (e.g. voting, protesting), given the unavailability of robust quantitative estimates of per-capita emission reductions associated with engagement in collective actions. Thus, it is possible that if participants had been presented with data on the relative effectiveness of collective engagement, the observed negative spillover could have been mitigated or reversed. Future work could attempt further quantifying the climate mitigation potential associated with collective actions, such as participating in the electoral process (9). Doing so would help future literacy interventions display the relative effectiveness of such actions compared to individual-level lifestyle behaviors (43).

Moreover, our study's collective-action measures primarily targeted governmental pathways (e.g. voting, protesting), whereas many current climate efforts also involve private-sector or nonprofit strategies, including corporate boycotts, shareholder advocacy, and initiatives like the Carbon Disclosure Project or the Science-Based Targets initiative. Because we did not include such actions, we cannot determine whether negative spillover would similarly occur for those private or community-level engagements. Future work should test a broader suite of collective options to account for contexts where government action is perceived as blocked. Nonetheless, individual actions can have effects beyond direct emissions reductions by shaping social norms, driving corporate reputational concerns, and fostering market demand for low-carbon products. These indirect pathways can, in some instances, lead to broader systemic changes, underscoring the potential importance of individual behaviors in complementing larger policy- or corporate-level efforts.

Furthermore, while we recommend not omitting collective-level actions in climate literacy interventions, we acknowledge that advocating for government-focused action could inadvertently alienate certain audiences if it signals partisan alignment. A tailored approach that highlights nonpartisan frames, community benefits, or cost-saving potential may help mitigate identity threats and sustain overall climate engagement across the political spectrum.

Finally, despite our efforts to assess the effects' durability by measuring the outcomes again one week post intervention, which balanced longitudinal testing with minimized attrition, this time frame is insufficient to capture even longer-term behavioral shifts. Future interventions could use longer follow-up windows (e.g. months or even years) and incorporate repeated “booster” exposures, social accountability strategies, goal-setting, or contextual cues to maintain and strengthen intervention effects over time.

We also encourage future research to investigate the relative effectiveness of the literacy interventions tested here and other theoretically based interventions, such as social norm messaging (44) or emotional appeals (4), at increasing accuracy about the mitigation potential of different climate actions and nudging commitments to the most effective behaviors.

Indeed, the field of environmental psychology has been increasingly advocating focusing on higher-impact behaviors in scholarship and interventions (27, 28). Promisingly, we found that behaviors such as taking one fewer flight, adopting one fewer dog, or eating lower-carbon meats had both high levels of carbon emissions reduction potential and behavioral plasticity (i.e. perceived ease of engagement). Thus, when it comes to individual lifestyle behaviors, we recommend that practitioners and scholars prioritize these actions when developing interventions in the United States.

Overall, our results suggest that evidence-based communication about the climate impact of individual actions can shift perceptions and commitments toward more impactful behaviors. Increasing climate literacy through the dissemination of accurate behavioral efficacy information is a low-cost and scalable strategy to guide consumers toward more sustainable decisions. This study contributes to the growing body of work investigating how information provision can facilitate sustainable behavior change and offers insights for policymakers, educators, and organizations seeking to empower individuals to take more effective climate action.

## Materials and methods

### Open science practices

All data and code are publicly available through the Open Science Framework.

Code, data, and preregistration available at: https://osf.io/fhyv6/? view_only=c891e0975d724293b6480767a8b56631

#### Participants

We recruited a sample of 3,946 Americans on Prolific. The data were collected during February and March of 2024. As preregistered, participants who failed to answer the attention check question correctly (i.e. “Please set the slider bar to 100 if you are still reading these questions”; *n* = 51) were excluded from the analysis. Overall, 3,895 participants (*M*_age_ = 41.48, SD_age_ =13.49; 2,080 [53%] women, 1,718 [44%] men, and 75 [2%] who identified as “other”; 2,021 [52%] Democrats, 869 [22%] Republicans, and 1,005 [26%] who identified as “other”) who passed the attention check were included in data analyses.

At time 2, 1 week posttreatment (*M* = 7 days posttreatment, SD = 1 day), a total of 3,131 participants from the original sample completed the follow-up survey (*M*_age_ = 42.39, SD_age_ = 13.46). The sample consisted of 1,633 (52%) women, 1,425 (46%) men, and 54 (2%) who identified as “other,” and 1,613 (52%) Democrats, 723 (23%) Republicans, and 795 (25%) who identified as “other.”

The study protocol was approved by the New York University Institutional Review Board, and informed consent was obtained from all participants.

#### Design and procedure

We used a between-subjects experimental design. After providing informed consent, participants rated 26 carbon emission-reducing behaviors (Table 1). Of these, 21 were individual-level proenvironmental behaviors, selected from a meta-analysis quantifying the carbon emission potential of a wide variety of climate mitigation behaviors (2). The additional five behaviors were collective climate actions. Participants were presented with these items in randomized order, and they were asked to rate the degree to which they can commit to engaging in each item, on a scale from 0 (“definitely not”) to 100 (“absolutely yes”). For each behavior, there was also an option to select “I already do this.” While the individual-level behaviors had quantifiable carbon emissions mitigation data (retrieved from 2), for the five collective behaviors we did not find quantifiable emissions reduction estimates (e.g. voting, donating to an environmental organization, etc.), given their indirect climate mitigation effects.

After the pretest commitment ratings, participants were assigned to one of three conditions: Control (no information), Information Only (action efficacy information), and Prediction (action efficacy information in a prediction-then-feedback format).

Participants in the Control condition read a distracter text from the novel “Great Expectations” by Charles Dickens.

**Fig. 7.**
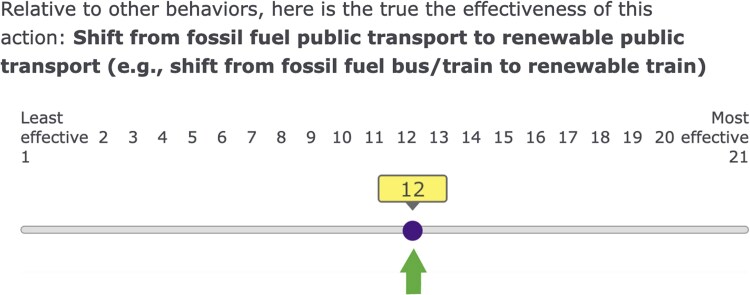
Screenshot of the survey material depicting the true feedback.

Participants in the Information-Only condition were shown an image depicting each of the 21 individual-level behaviors (in randomized order) positioned on a scale ranging from least effective to most effective, highlighting the true relative efficacy of that behavior (Fig. 7).

**Fig. 8.**
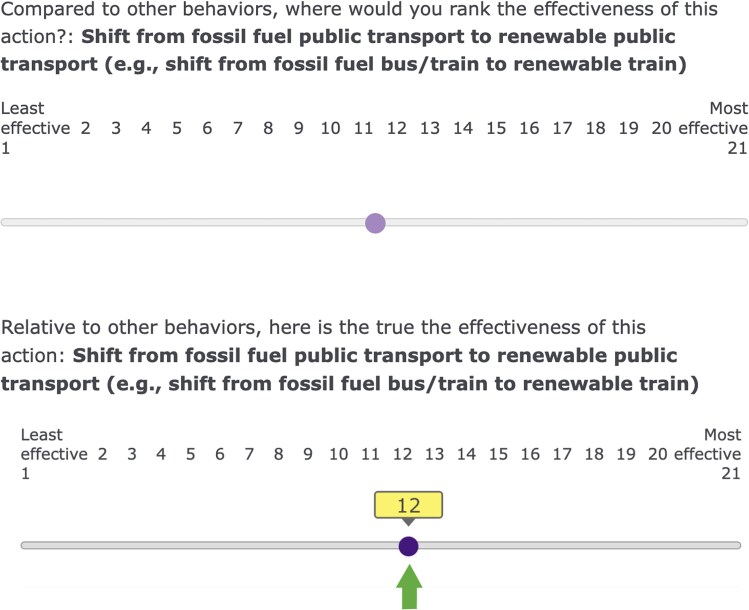
Screenshot of the survey material depicting the ranking prediction task and true feedback.

Participants in the Prediction condition were first asked to predict, relative to the other behaviors, where they would rank the effectiveness of each individual-level behavior, before being shown the true effectiveness ranking of that behavior (Fig. 8).

Following this procedure, all participants again rated their posttest commitment levels for each of the 26 behaviors. Participants then rated how effective they perceived each behavior to be in terms of carbon mitigation potential, on a scale from 0 (extremely ineffective) to 100 (extremely effective). Next, participants rated each behavior on how easy or difficult it would be for them to engage in each behavior, on a scale from 0 (extremely difficult) to 100 (extremely easy). Finally, we collected demographic information, including gender, age, education, political party, political ideology, income, and socioeconomic status. We also asked participants about the degree to which they think climate change poses a threat to human civilization and their perception on what percent of climate scientists agree that human-caused climate change is happening. Participants were then debriefed and compensated for their participation.

### Materials

Twenty-six behaviors used in this study are summarized in Table 1.

### Measures

#### Independent variables

##### Ranking (Prediction condition)

Participants in this condition were asked for each behavior, “Compared to other behaviors, where would you rank the effectiveness of this action?” They were then asked to set a slider to a position from 1 (“least effective” to 21 “most effective”). Immediately after setting the slider, they were shown “Relative to other behaviors, here is the true effectiveness of this action,” alongside an image of the slider with the true relative effectiveness highlighted with a green arrow. Each of the 21 behaviors only had one correct position on the slider, reflecting its relative ranking compared with the other behaviors, according to a meta-analysis (2).

##### Information-Only

Participants in the Information-only condition were only shown these images revealing the true relative effectiveness for each behavior.

##### Control

Participants in the control condition were shown an excerpt from the Charles Dickens novel “Great Expectations.”

#### Dependent variables

##### Commitments (pre- and posttest)

For each behavior (in randomized order), we asked, “Can you commit to this action?” followed by the behavior. Participants responded on a scale from 0 (“definitely not”) to 100 (“absolutely yes”). There was also an option to select “I already do this.” Participants who selected “I already do this” were excluded from the relevant analyses.

##### Effectiveness (posttest)

Participants were told, “You will now be asked to rate the carbon emissions-reducing behaviors on the degree to which you perceive them to be effective in terms of carbon mitigation potential.” Then, for each behavior, they were asked, “How effective in terms of carbon mitigation potential do you perceive this action to be?” on a scale from 0 (“extremely ineffective”) to 100 (“extremely effective”).

##### Plasticity (posttest)

Participants were told, “You will now be asked to rate the carbon emissions-reducing behaviors on how easy or difficult it would be for you to do this action.” They were then asked for each behavior, “How difficult or easy would it be for you to do this action?” on a scale from 0 (“extremely difficult”) to 100 (“extremely easy”).

##### Demographics

Participants reported their gender (male; female; prefer not to say; nonbinary/third gender/other), age, and years of formal education categorized as 0–6 years (up to grade school/elementary school), 7–12 years (up to high school), 13–16 years (college/undergraduate university/certificate training), and more than 17 years (doctorate degree, medical degree, etc.), or “prefer not to answer.” We assessed political party identification (Democrat; Republican; Other) and political orientation for both social (e.g. health care, education) and economic (e.g. taxes) issues, with response scales ranging from “extremely liberal/left-wing” to “extremely conservative/right-wing.” Participants also indicated their total yearly family/household income using the following brackets: less than $10,000; $10,000–$14,999; $15,000–$24,999; $25,000–$49,999; $50,000–$99,999; $100,000–$149,999; $150,000–$199,999; $200,000 or more; or “prefer not to respond.” Socioeconomic status was measured using the MacArthur Scale of Subjective Social Status, where participants chose the rung of a ladder that best represented their standing relative to other people in the United States.

In addition, we asked participants two climate-related questions. The first measured perceived threat by asking: “To what degree do you think climate change poses a threat to human civilization?” Responses were given on a scale from 0 (“not at all”) to 100 (“extremely”). The second assessed perceived scientific consensus: “To the best of your knowledge, what percentage of climate scientists have concluded that human-caused climate change is happening?” Participants indicated a value on a sliding scale from 0 to 100.

## Supplementary Material

pgaf191_Supplementary_Data
