# Supplementary material for: Climate action literacy interventions increase commitments to more effective mitigation behaviors
Source: PNAS Nexus. 2025 Jun 9;4(6):pgaf191. doi: 10.1093/pnasnexus/pgaf191 (PMC12198959; doi:10.1093/pnasnexus/pgaf191)
Supplement: pgaf191_Supplementary_Data [file pgaf191_supplementary_data.zip › PNASNEXUS-PNASNEXUS-2025-00193R-s01.pdf]

Supplementary Materials for:

Climate Action Literacy Interventions Increase Commitments to More Effective Mitigation Behaviors

Corresponding author: Danielle Goldwert, danielle.goldwert@nyu.edu

Contents

1 Time 1 **3**

1.1 Prediction Error Across Demographic Groups . . . 3

1.2 Hypothesis 1 . . . 9

1.3 Hypothesis 2 . . . 11

1.4 Hypothesis 3 . . . 14

1.4.1 Political Affiliation as a Moderator . . . 17

1.5 Hypothesis 4 . . . 18

1.6 Exploratory . . . 20

2 Time 2 **22**

2.1 Hypothesis 1 . . . 22

2.2 Hypothesis 2 . . . 24

2.3 Hypothesis 3 . . . 27

2.3.1 Political Affiliation as a Moderator . . . 30

2.4 Hypothesis 4 . . . 31

The following sections provide the results from our mixed effects models, calculated using R and directly piped into this document.

1 Time 1

1.1 Prediction Error Across Demographic Groups

Table S1

Variable	*SS*	*df*	*F*	*Pr(>F)*
Item	1156903.43	20.0	2777.67	<.001
Politics	2651.20	1.0	127.31	<.001
Item:Politics	640.76	20.0	1.54	0.058
Age	183.09	1.0	8.79	0.003
Item:Age	1070.62	20.0	2.57	<.001
Income	29.07	1.0	1.40	0.237
Item:Income	236.91	20.0	0.57	0.936
Education	0.77	1.0	0.04	0.848
Item:Education	695.13	20.0	1.67	0.031
Gender	132.63	1.0	6.37	0.012
Item:Gender	893.88	20.0	2.15	0.002
Residual	560,839.24	26931.0		

Note. The ANOVA model includes behavior item, demographics (political affiliation, age, income, education, and gender), and their interactions. Prediction error served as the dependent variable."

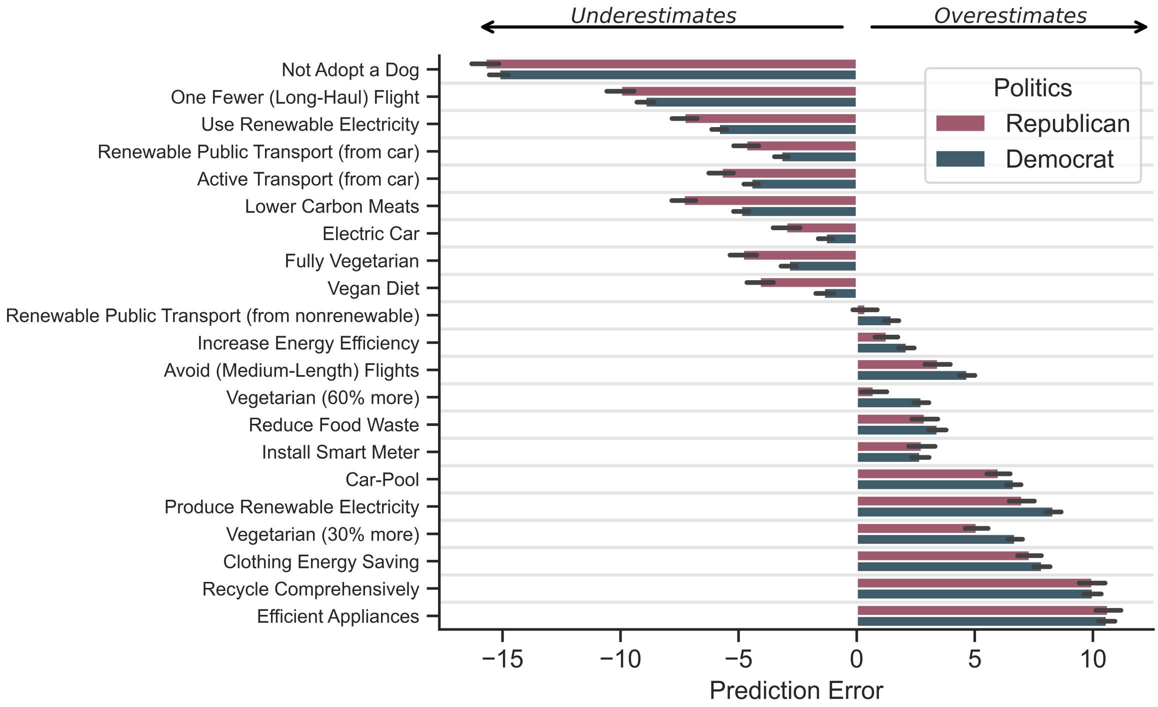

Figure S1. Prediction errors (i.e., the difference between the predicted and actual rank) associated with each of the 21 individual-level climate-relevant behaviors assessed, split by political party (Republican, Democrat). Error bars represent 95% Confidence Intervals.

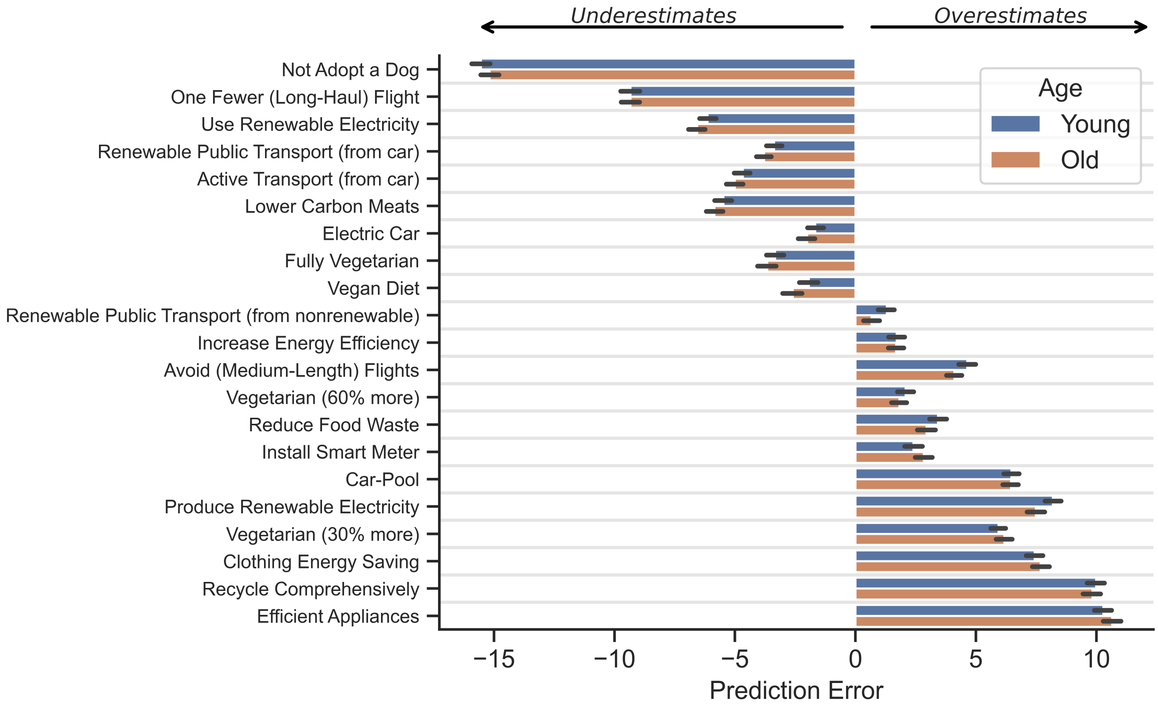

Figure S2. Prediction errors (i.e., the difference between the predicted and actual rank) associated with each of the 21 individual-level climate-relevant behaviors assessed, split by age (young, old). Error bars represent 95% Confidence Intervals.

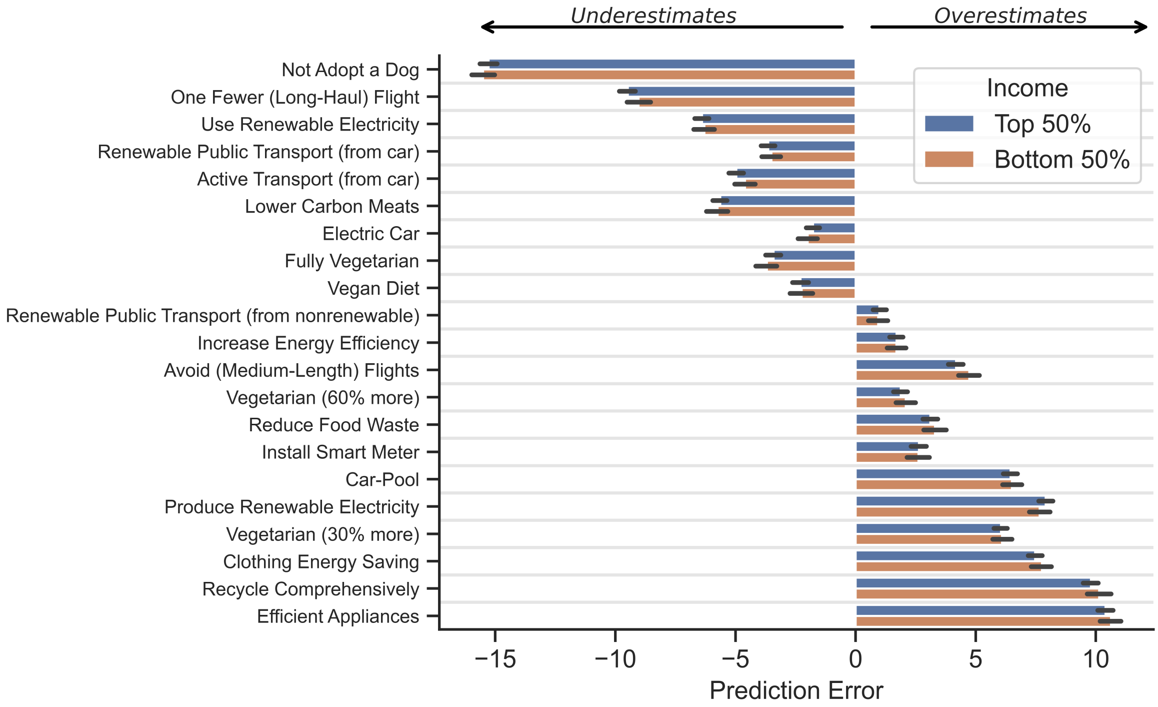

Figure S3. Prediction errors (i.e., the difference between the predicted and actual rank) associated with each of the 21 individual-level climate-relevant behaviors assessed, split by income (top 50%, bottom 50%). Error bars represent 95% Confidence Intervals.

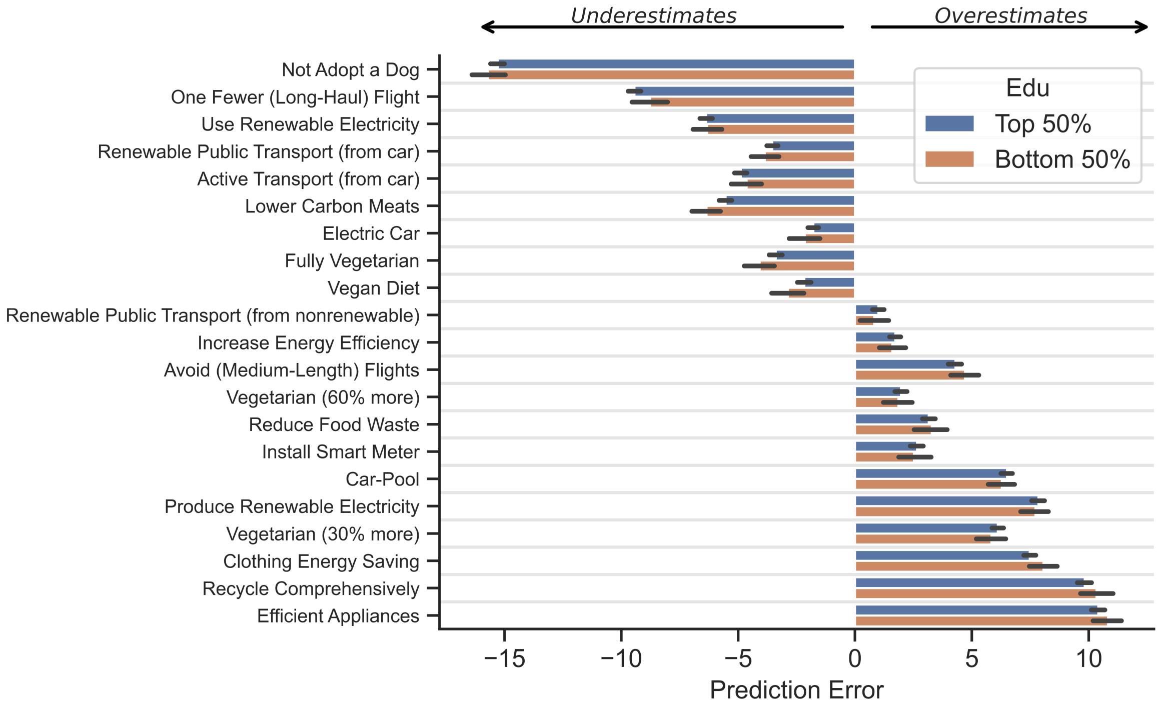

Figure S4. Prediction errors (i.e., the difference between the predicted and actual rank) associated with each of the 21 individual-level climate-relevant behaviors assessed, split by education level (top 50%, bottom 50%). Error bars represent 95% Confidence Intervals.

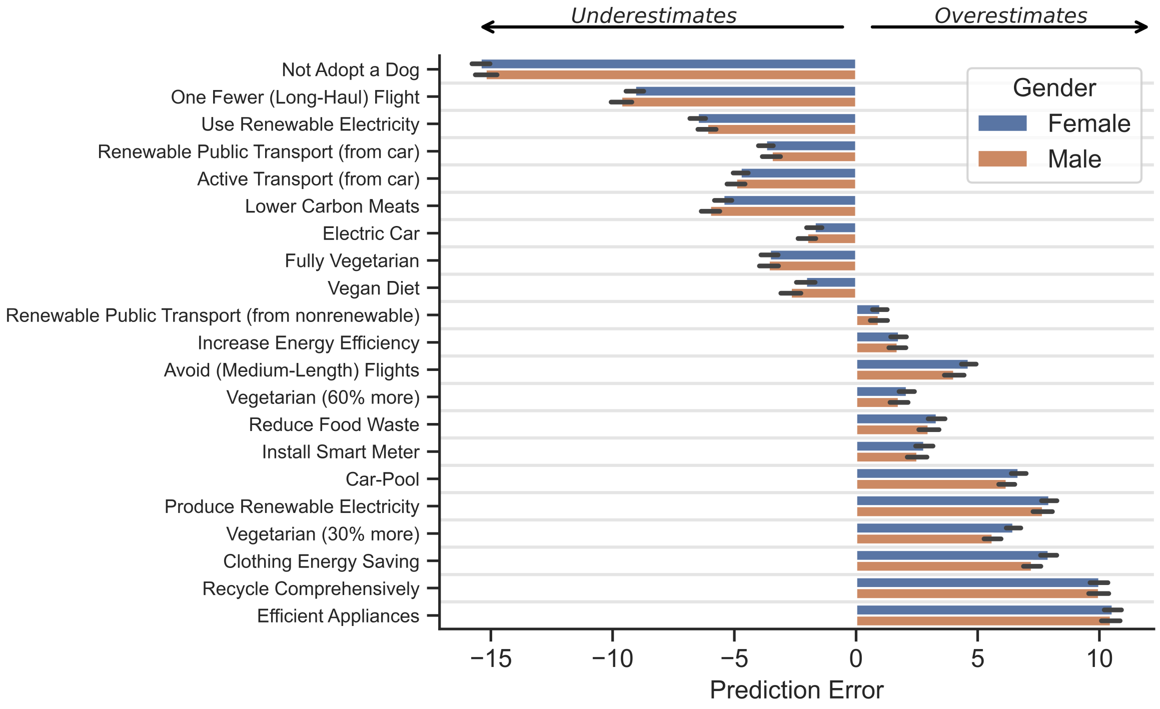

Figure S5. Prediction errors (i.e., the difference between the predicted and actual rank) associated with each of the 21 individual-level climate-relevant behaviors assessed, split by gender (female, male). Error bars represent 95% Confidence Intervals.

1.2 Hypothesis 1

Table S2. Results of the Linear Mixed-Effects Model Predicting Perceived Efficacy.

Condition	Estimate	*SE*	*t*	*d*	*p*	95% CI
(Intercept)	58.16	0.55	105.55	2.70	<.001	[57.08, 59.24]
Information	-12.49	0.78	-16.05	-0.58	<.001	[-14.01, -10.96]
Prediction	-11.17	0.78	-14.33	-0.52	<.001	[-12.69, -9.64]
RealEff	-4.31	0.28	-15.37	-0.20	<.001	[-4.86, -3.76]
Information:RealEff	16.52	0.40	41.72	0.77	<.001	[15.74, 17.29]
Prediction:RealEff	15.56	0.40	39.25	0.72	<.001	[14.78, 16.34]

Note. The model includes condition (Control, Information, Prediction), actual efficacy (RealEff), and their interaction as fixed effects, with random intercepts for participants. Perceived efficacy served as the dependent variable.

Table S3. Results of the Linear Mixed-Effects Model Predicting Perceived Efficacy with Reordered Condition Levels

Condition	Estimate	*SE*	*t*	*d*	*p*	95% CI
(Intercept)	47.00	0.55	85.36	2.18	<.001	[45.92, 48.08]
aControl	11.17	0.78	14.33	0.52	<.001	[9.64, 12.69]
Information	-1.32	0.78	-1.70	-0.06	.089	[-2.85, 0.20]
RealEff	11.25	0.28	40.16	0.52	<.001	[10.70, 11.80]
aControl:RealEff	-15.56	0.40	-39.25	-0.72	<.001	[-16.34, -14.78]
Information:RealEff	0.96	0.40	2.42	0.04	.015	[0.18, 1.73]

Note. The model includes condition (Prediction, Control, Information), actual efficacy (RealEff), and their interaction as fixed effects, with random intercepts for participants. Perceived efficacy served as the dependent variable. The Prediction condition serves as the reference group.

1.3 Hypothesis 2

Table S4. Results of the Linear Mixed-Effects Model Predicting Change in Commitments from Pre-Test to Post-Test

Condition	Estimate	*SE*	*t*	*d*	*p*	95% CI
(Intercept)	-1.45	0.31	-4.67	-0.08	<.001	[-2.07, -0.84]
Information	-1.90	0.44	-4.31	-0.10	<.001	[-2.76, -1.03]
Prediction	-3.16	0.44	-7.17	-0.16	<.001	[-4.03, -2.30]
RealEff	0.88	0.29	3.06	0.05	.002	[0.31, 1.44]
Information:RealEff	3.78	0.41	9.32	0.20	<.001	[2.99, 4.58]
Prediction:RealEff	4.08	0.41	10.05	0.21	<.001	[3.29, 4.88]

Note. The model includes condition (Control, Information, Prediction), actual efficacy (RealEff), and their interaction as fixed effects, with random intercepts for participants. Change in commitments from pre-test to post-test served as the dependent variable.

Table S5. Results of the Linear Mixed-Effects Model Predicting Change in Commitments from Pre-Test to Post-Test with Reordered Condition Levels

Condition	Estimate	*SE*	*t*	*d*	*p*	95% CI
(Intercept)	-4.62	0.31	-14.79	-0.24	<.001	[-5.23, -4.00]
aControl	3.16	0.44	7.17	0.16	<.001	[2.30, 4.03]
Information	1.26	0.44	2.86	0.07	.004	[0.40, 2.13]
RealEff	4.96	0.29	17.22	0.26	<.001	[4.39, 5.52]
aControl:RealEff	-4.08	0.41	-10.05	-0.21	<.001	[-4.88, -3.29]
Information:RealEff	-0.30	0.41	-0.74	-0.02	.460	[-1.10, 0.50]

Note. The model includes condition (Control, Information, Prediction), actual efficacy (RealEff), and their interaction as fixed effects, with random intercepts for participants. Change in commitments from pre-test to post-test served as the dependent variable. The Prediction condition serves as the reference group.

Table S6. Results of the Mediation Analysis Testing the Indirect Effect of Condition on Change in Commitments via Perceived Efficacy

Effect	Estimate	95% CI Lower	95% CI Upper	p-value
ACME	0.04407	0.00213	0.08649	.040
ADE	-0.11207	-0.47605	0.25949	.626
Total Effect	-0.06800	-0.44852	0.30647	.794
Prop. Mediated	-0.64813	-2.61526	3.60223	.806

Note. The mediation analysis examined whether perceived efficacy mediates the effect of condition (Experimental [combined Prediction and Information] vs. Control) on the change in commitments from pre-test to post-test. The mediator model regressed perceived efficacy on condition, and the outcome model regressed commitment change on both condition and perceived efficacy. Bootstrapping with 1,000 simulations was used to estimate confidence intervals for the indirect effect.

1.4 Hypothesis 3

Table S7. Results of the Mixed-Effects Model Predicting Change in Commitments from Pre-Test to Post-Test Based on Prediction Error in the Prediction Condition

Condition	Estimate	*SE*	*t*	*d*	*p*	95% CI
(Intercept)	-0.96	0.25	-3.84	-0.05	<.001	[-1.45, -0.47]
poly(sPE, 2)1	-262.57	25.60	-10.26	-12.36	<.001	[-312.74, -212.40]
poly(sPE, 2)2	-115.78	25.78	-4.49	-5.45	<.001	[-166.32, -65.25]

Note. The model includes a quadratic term for prediction error to capture potential non-linear effects and a random intercept for participants. The analysis is restricted to participants in the Prediction condition, with change in commitments from pre-test to post-test serving as the dependent variable.

Table S8. Results of the Linear Mixed-Effects Model Predicting Change in Commitments from Pre-Test to Post-Test Based on Prediction Error in the Prediction Condition

Condition	Estimate	*SE*	*t*	*d*	*p*	95% CI
(Intercept)	-0.81	0.25	-3.24	-0.04	.001	[-1.30, -0.32]
sPE	-0.20	0.02	-10.14	-0.01	<.001	[-0.24, -0.16]

Note. The model includes prediction error as a fixed effect and a random intercept for participants. The analysis is restricted to participants in the Prediction condition, with change in commitments from pre-test to post-test serving as the dependent variable.

Table S9. Results of the Linear Mixed-Effects Model Predicting Change in Commitments from Pre-Test to Post-Test Based on Group

Condition	Estimate	*SE*	*t*	*d*	*p*	95% CI
(Intercept)	-0.79	0.22	-3.54	-0.04	<.001	[-1.22, -0.35]
PEAccurate	0.56	0.65	0.86	0.03	.390	[-0.72, 1.84]
PEInformation	0.99	0.31	3.16	0.05	.002	[0.37, 1.60]
PEOverestimate	-1.34	0.34	-3.91	-0.07	<.001	[-2.00, -0.67]
PEUnderestimate	1.29	0.35	3.72	0.07	<.001	[0.61, 1.97]

Note. The model includes group (Control, Information, Accurate, Overestimate, Underestimate) as a fixed effect and random intercepts for participants. Change in commitments from pre-test to post-test served as the dependent variable.

1.4.1 Political Affiliation as a Moderator

Table S10. Results of the Linear Mixed-Effects Model Predicting Change in Commitments from Pre-Test to Post-Test Based on Prediction Error and Political Affiliation in the Prediction Condition

Condition	Estimate	*SE*	*t*	*d*	*p*	95% CI
(Intercept)	-0.78	0.35	-2.21	-0.04	.028	[-1.48, -0.09]
sPE	-0.26	0.03	-9.08	-0.01	<.001	[-0.31, -0.20]
PoliticsRepublicans	1.36	0.62	2.17	0.06	.030	[0.13, 2.58]
sPE:PoliticsRepublicans	0.14	0.05	2.92	0.01	.004	[0.04, 0.23]

Note. The model includes prediction error, political affiliation (Democrats, Republicans), and their interaction as fixed effects, with random intercepts for participants. The analysis is restricted to participants in the Prediction condition, with change in commitments from pre-test to post-test serving as the dependent variable.

1.5 Hypothesis 4

Table S11. Results of Tukey's Honest Significant Difference (HSD) Test for Pairwise Comparisons of Change in Collective Action Commitments Across Conditions

Group 1	Group 2	Mean Difference	*p-adj*	*lower*	*upper*
Control	Information	-1.202	$<.001$	-1.45	-0.96
Control	Prediction	-1.76	$<.001$	-2.01	-1.52
Information	Prediction	-0.56	$<.001$	-0.81	-0.32

Note. Tukey's HSD test was conducted to compare the mean change in commitments to collective actions between all pairs of conditions.

Table S12. Results of the Mediation Analysis Testing the Indirect Effect of Experimental Condition on Change in Commitments to Collective Behaviors via Perceived Efficacy

Effect	Estimate	95% CI Lower	95% CI Upper	p-value
ACME	0.01580	0.00325	0.02877	.010
ADE	-1.50019	-1.65318	-1.33662	<.001
Total Effect	-1.48439	-1.63991	-1.32173	<.001
Prop. Mediated	-0.01064	-0.01994	-0.00211	.010

Note. The mediation analysis examined whether perceived efficacy mediates the effect of condition (Experimental [combined Prediction and Information] vs. Control) on the change in commitments to collective behaviors. The mediator model regressed perceived efficacy on condition, and the outcome model regressed change in commitments on both condition and perceived efficacy. Bootstrapping with 1,000 simulations was used to estimate confidence intervals for the indirect effect.

1.6 Exploratory

Table S13. Results of the Linear Mixed-Effects Model Predicting Pre-Test Individual Action Commitments Based on Perceived Efficacy and Plasticity in the Control Condition

Condition	Estimate	*SE*	*t*	*d*	*p*	95% CI
(Intercept)	6.99	0.51	13.59	0.32	<.001	[5.99, 8.00]
PLAS	0.65	0.00	131.94	0.03	<.001	[0.64, 0.66]
EFF	0.15	0.01	21.71	0.01	<.001	[0.13, 0.16]

Note. The model includes perceived efficacy, plasticity, and their interaction as fixed effects, with random intercepts for participants. The analysis is restricted to participants in the Control condition, with pre-test commitments to individual level behaviors serving as the dependent variable.

Table S14. Results of the Linear Mixed-Effects Model Predicting Pre-Test Collective Action Commitments Based on Perceived Efficacy and Plasticity in the Control Condition

Condition	Estimate	*SE*	*t*	*d*	*p*	95% CI
(Intercept)	6.38	0.74	8.57	0.32	<.001	[4.92, 7.84]
PLAS_OL	0.40	0.01	38.56	0.02	<.001	[0.38, 0.42]
EFF_OL	0.44	0.01	33.69	0.02	<.001	[0.41, 0.46]

Note. The model includes perceived efficacy, plasticity, and their interaction as fixed effects, with random intercepts for participants. The analysis is restricted to participants in the Control condition, with pre-test commitments to collective level behaviors serving as the dependent variable.

2 Time 2

The following results compares participants' pre-intervention measures with one week follow-up.

2.1 Hypothesis 1

Table S15. Results of the Linear Mixed-Effects Model Predicting Perceived Efficacy.

Condition	Estimate	*SE*	*t*	*d*	*p*	95% CI
(Intercept)	56.32	0.64	87.88	2.50	<.001	[55.06, 57.58]
Information	-7.48	0.91	-8.19	-0.33	<.001	[-9.27, -5.69]
Prediction	-8.65	0.90	-9.56	-0.38	<.001	[-10.42, -6.88]
RealEff	-3.05	0.29	-10.38	-0.14	<.001	[-3.63, -2.48]
Information:RealEff	7.97	0.42	19.00	0.35	<.001	[7.15, 8.79]
Prediction:RealEff	9.08	0.42	21.85	0.40	<.001	[8.27, 9.90]

Note. The model includes condition (Control, Information, Prediction), actual efficacy (RealEff), and their interaction as fixed effects, with random intercepts for participants. Perceived efficacy served as the dependent variable.

Table S16. Results of the Linear Mixed-Effects Model Predicting Perceived Efficacy with Reordered Condition Levels

Condition	Estimate	*SE*	*t*	*d*	*p*	95% CI
(Intercept)	47.67	0.64	74.61	2.12	<.001	[46.42, 48.92]
aControl	8.65	0.90	9.56	0.38	<.001	[6.88, 10.42]
Information	1.17	0.91	1.28	0.05	.201	[-0.62, 2.96]
RealEff	6.03	0.29	20.53	0.27	<.001	[5.45, 6.60]
aControl:RealEff	-9.08	0.42	-21.85	-0.40	<.001	[-9.90, -8.27]
Information:RealEff	-1.11	0.42	-2.65	-0.05	.008	[-1.93, -0.29]

Note. The model includes condition (Prediction, Control, Information), actual efficacy (RealEff), and their interaction as fixed effects, with random intercepts for participants. Perceived efficacy served as the dependent variable. The Prediction condition serves as the reference group.

2.2 Hypothesis 2

Table S17. Results of the Linear Mixed-Effects Model Predicting Change in Commitments from Pre-Test to Follow-Up

Condition	Estimate	*SE*	*t*	*d*	*p*	95% CI
(Intercept)	-2.50	0.43	-5.74	-0.10	<.001	[-3.35, -1.65]
Information	0.38	0.62	0.61	0.01	.542	[-0.84, 1.59]
Prediction	0.60	0.61	0.97	0.02	.330	[-0.61, 1.80]
RealEff	0.84	0.39	2.14	0.03	.032	[0.07, 1.61]
Information:RealEff	0.35	0.56	0.62	0.01	.536	[-0.75, 1.45]
Prediction:RealEff	0.69	0.55	1.25	0.03	.210	[-0.39, 1.78]

Note. The model includes condition (Control, Information, Prediction), actual efficacy (RealEff), and their interaction as fixed effects, with random intercepts for participants. Change in commitments from pre-test to follow-up served as the dependent variable.

Table S18. Results of the Linear Mixed-Effects Model Predicting Change in Commitments from Pre-Test to Follow-Up with Reordered Condition Levels

Condition	Estimate	*SE*	*t*	*d*	*p*	95% CI
(Intercept)	-1.90	0.43	-4.38	-0.07	<.001	[-2.75, -1.05]
aControl	-0.60	0.61	-0.97	-0.02	.330	[-1.80, 0.61]
Information	-0.22	0.62	-0.36	-0.01	.722	[-1.43, 0.99]
RealEff	1.54	0.39	3.93	0.06	<.001	[0.77, 2.30]
aControl:RealEff	-0.69	0.55	-1.25	-0.03	.210	[-1.78, 0.39]
Information:RealEff	-0.35	0.56	-0.62	-0.01	.534	[-1.44, 0.75]

Note. The model includes condition (Control, Information, Prediction), actual efficacy (RealEff), and their interaction as fixed effects, with random intercepts for participants. Change in commitments from pre-test to follow-up served as the dependent variable. The Prediction condition serves as the reference group.

Table S19. Results of the Mediation Analysis Testing the Indirect Effect of Condition on Change in Commitments via Perceived Efficacy

Effect	Estimate	95% CI Lower	95% CI Upper	p-value
ACME	0.04407	0.00063	0.08713	.046
ADE	-0.11207	-0.49121	0.26067	.572
Total Effect	-0.06800	-0.44833	0.31562	.716
Prop. Mediated	-0.64813	-3.30687	2.92792	.738

Note. The mediation analysis examined whether perceived efficacy mediates the effect of condition (Experimental [combined Prediction and Information] vs. Control) on the change in commitments from pre-test to follow-up. The mediator model regressed perceived efficacy on condition, and the outcome model regressed commitment change on both condition and perceived efficacy. Bootstrapping with 1,000 simulations was used to estimate confidence intervals for the indirect effect.

2.3 Hypothesis 3

Table S20. Results of the Linear Mixed-Effects Model Predicting Change in Commitments from Pre-Test to Follow-Up Based on Prediction Error in the Prediction Condition

Condition	Estimate	*SE*	*t*	*d*	*p*	95% CI
(Intercept)	-0.72	0.32	-2.28	-0.03	.023	[-1.34, -0.10]
poly(sPE, 2)1	-162.89	31.73	-5.13	-6.44	<.001	[-225.09, -100.69]
poly(sPE, 2)2	109.06	32.23	3.38	4.31	<.001	[45.89, 172.23]

Note. The model includes a quadratic term for prediction error to capture potential non-linear effects and a random intercept for participants. The analysis is restricted to participants in the Prediction condition, with change in commitments from pre-test to follow-up serving as the dependent variable.

Table S21. Results of the Linear Mixed-Effects Model Predicting Change in Commitments from Pre-Test to Follow-Up Based on Prediction Error in the Prediction Condition

Condition	Estimate	*SE*	*t*	*d*	*p*	95% CI
(Intercept)	-0.92	0.26	-3.57	-0.03	$<.001$	[-1.43, -0.42]
sPE	-0.19	0.02	-9.87	-0.01	$<.001$	[-0.23, -0.16]

Note. The model includes prediction error as a fixed effect and a random intercept for participants. The analysis is restricted to participants in the Prediction condition, with change in commitments from pre-test to follow-up serving as the dependent variable.

Table S22. Results of the Linear Mixed-Effects Model Predicting Change in Commitments from Pre-Test to Follow-Up Based on Group

Condition	Estimate	*SE*	*t*	*d*	*p*	95% CI
(Intercept)	-1.85	0.31	-5.91	-0.07	<.001	[-2.47, -1.24]
PEAccurate	1.29	0.88	1.47	0.05	.142	[-0.43, 3.02]
PEInformation	0.64	0.45	1.44	0.03	.149	[-0.23, 1.52]
PEOverestimate	0.63	0.48	1.32	0.02	.187	[-0.31, 1.57]
PEUnderestimate	1.63	0.48	3.37	0.06	<.001	[0.68, 2.58]

Note. The model includes group (Control, Information, Accurate, Overestimate, Underestimate) as a fixed effect and random intercepts for participants. Change in commitments from pre-test to follow-up served as the dependent variable.

2.3.1 Political Affiliation as a Moderator

Table S23. Results of the Linear Mixed-Effects Model Predicting Change in Commitments from Pre-Test to Follow-Up Based on Prediction Error and Political Affiliation in the Prediction Condition

Condition	Estimate	*SE*	*t*	*d*	*p*	95% CI
(Intercept)	-1.12	0.44	-2.56	-0.04	.011	[-1.98, -0.26]
sPE	-0.14	0.03	-4.00	-0.01	<.001	[-0.21, -0.07]
PoliticsRepublicans	1.71	0.77	2.21	0.07	.027	[0.20, 3.23]
sPE:PoliticsRepublicans	-0.01	0.06	-0.20	-0.00	.844	[-0.13, 0.10]

Note. The model includes prediction error, political affiliation (Democrats, Republicans), and their interaction as fixed effects, with random intercepts for participants. The analysis is restricted to participants in the Prediction condition, with change in commitments from pre-test to follow-up serving as the dependent variable.

2.4 Hypothesis 4

Table S24. Results of the Mediation Analysis Testing the Indirect Effect of Experimental Condition on Change in Commitments to Collective Behaviors via Perceived Efficacy

Effect	Estimate	95% CI Lower	95% CI Upper	p-value
ACME	0.00010	-0.01108	0.00987	.956
ADE	0.12862	-0.13923	0.38220	.358
Total Effect	0.12873	-0.13620	0.38159	.358
Prop. Mediated	0.00080	-0.43914	0.21922	.998

Note. The mediation analysis examined whether perceived efficacy mediates the effect of condition (Experimental [combined Prediction and Information] vs. Control) on the change in commitments to collective behaviors. The mediator model regressed perceived efficacy on condition, and the outcome model regressed change in commitments on both condition and perceived efficacy. Bootstrapping with 1,000 simulations was used to estimate confidence intervals for the indirect effect.
