# Supplementary material for: Climate action literacy interventions increase commitments to more effective mitigation behaviors
Source: PNAS Nexus. 2025 Jun 9;4(6):pgaf191. doi: 10.1093/pnasnexus/pgaf191 (PMC12198959; doi:10.1093/pnasnexus/pgaf191)
Supplement: pgaf191_Supplementary_Data [file pgaf191_supplementary_data.zip › PNASNEXUS-PNASNEXUS-2025-00193R-s02.pdf]

ClimateActions

Survey Flow

Standard: Consent (1 Question)
Standard: ProlificID (1 Question)

EmbeddedData
aidValue will be set from Panel or URL.

Standard: Commitment_InstructionsPRE (1 Question)
Standard: Commitment_PRE (27 Questions)

BlockRandomizer: 1 - Evenly Present Elements

EmbeddedData
C = Experimental
EmbeddedData
C = ActiveControl
EmbeddedData
C = Control

Branch: New Branch
If
If C Is Equal to Experimental

Standard: Exp_ Ranking instructions (1 Question)

Branch: New Branch
If
If C Is Equal to Experimental

BlockRandomizer: 21 - Evenly Present Elements

Standard: Appliance_exp (2 Questions)
Standard: Recycle_exp (2 Questions)
Standard: Clothing_exp (2 Questions)
Standard: Vegetarian1_exp (2 Questions)
Standard: ProduceRElectric_exp (2 Questions)
Standard: CarPool_exp (2 Questions)
Standard: SmartMeter_exp (2 Questions)
Standard: ReduceWaste_exp (2 Questions)
Standard: Vegetarian2_exp (2 Questions)
Standard: LessFlight1_exp (2 Questions)
Standard: EnergyEff_exp (2 Questions)
Standard: PublicTransport1_exp (2 Questions)
Standard: Vegan_exp (2 Questions)
Standard: Vegetarian3_exp (2 Questions)
Standard: ElectricCar_exp (2 Questions)
Standard: Meats_exp (2 Questions)
Standard: PublicTransport2_exp (2 Questions)

Standard: ActiveTransport_exp (2 Questions)
Standard: UseRElectric_exp (2 Questions)
Standard: LessFlight2_exp (2 Questions)
Standard: Adopt_exp (2 Questions)

Branch: New Branch
If
If C Is Equal to ActiveControl

Standard: Actcontrol_ Ranking Instructions (1 Question)

Branch: New Branch
If
If C Is Equal to ActiveControl

BlockRandomizer: 21 - Evenly Present Elements

Standard: Appliance_control (1 Question)
Standard: Recycle_control (1 Question)
Standard: Clothing_control (1 Question)
Standard: Vegetarian1_control (1 Question)
Standard: ProduceRElectric_control (1 Question)
Standard: CarPool_control (1 Question)
Standard: SmartMeter_control (1 Question)
Standard: ReduceWaste_control (1 Question)
Standard: Vegetarian2_control (1 Question)
Standard: LessFlight1_control (1 Question)
Standard: EnergyEff_control (1 Question)
Standard: PublicTransport1_control (1 Question)
Standard: Vegan_control (1 Question)
Standard: Vegetarian3_control (1 Question)
Standard: ElectricCar_control (1 Question)
Standard: Meats_control (1 Question)
Standard: PublicTransport2_control (1 Question)
Standard: ActiveTransport_control (1 Question)
Standard: UseRElectric_control (1 Question)
Standard: LessFlight2_control (1 Question)
Standard: Adopt_control (1 Question)

Branch: New Branch
If
If C Is Equal to Control

Standard: Control_distracter (3 Questions)

Standard: Commitment_InstructionsPOST (1 Question)
Standard: Commitment_POST (26 Questions)

Standard: Effectiveness_InstructionsPOST (1 Question)
Standard: Effectiveness_POST (26 Questions)
Standard: Plasticity_InstructionsPOST (1 Question)
Standard: Plasticity_POST (26 Questions)
Standard: Demographics (13 Questions)

EndSurvey: Advanced

Page Break

Start of Block: Consent

Q1

Online Consent Form for IRB-FY2024-8662 You have been invited to take part in a research study to learn more about people's evaluation of information. We are interested how information about the effectiveness of environmental impacts relates to attitudes toward combatting climate change. Participants will be randomly assigned to see different levels of information about the effectiveness of pro-environmental behaviors. This study will be conducted by Madalina Vlasceanu, GSAS - Graduate School of Arts and Science, New York University. If you agree to be in this study, you will be asked to do the following: Complete a questionnaire assessing your judgments on specific carbon emissions-reducing behaviors. Complete demographic questions. You will be randomly assigned to one of three groups which will affect the the information given to you to read regarding climate change. Participation in this study will take about 10 minutes. You will be compensated $1.75 for completion in the survey via Prolific. There are no known risks associated with your participation in this research beyond those of everyday life. Although you will receive no direct benefits, this research may help the investigator understand how people learn information. Confidentiality of your research records will be strictly maintained by assigning unique, confidential identification number codes to your responses. Information not containing identifiers may be used in future research, shared with other researchers, or placed in a data repository without your additional consent. Participation in this study is voluntary. You may refuse to participate or withdraw at any time without penalty. Payment will not be sent if you do not answer all questions on the survey. If there is anything about the study or your participation that is unclear or that you do not understand, if you have questions or wish to report a research-related problem, you may contact Madalina Vlasceanu at vlasceanu@nyu.edu, 6 Washington Place, New York, NY 10003. For questions about your rights as a research participant, you may contact the Institutional Review Board (IRB), New York University, 665 Broadway, Suite 804, New York, New York, 10012 at (212) 998-4808 or ask.humansubjects@nyu.edu. Please reference the study # (IRB-FY2024-8662) when contacting the IRB. Clicking below serves as your consent to participate in the study. If you choose not to participate you may simply close this window.

End of Block: Consent

Start of Block: ProlificID

prolific Please provide your unique Prolific ID

____

End of Block: ProlificID

Start of Block: Commitment_InstructionsPRE

Instructions In the following survey, you will be asked to rate a series of carbon emissions-reducing behaviors on the degree to which you can commit to them.

End of Block: Commitment_InstructionsPRE

Start of Block: Commitment_PRE

Appliance_C1 Can you commit to this action?: **Use more efficient appliances (e.g., change your lightbulbs)**

	Definitely not	Absolutely yes	I already do this

0 10 20 30 40 50 60 70 80 90 100

()	

Recycle_C1 Can you commit to this action?: **Comprehensively recycle for at least one year**

	Definitely not	Absolutely yes	I already do this

0 10 20 30 40 50 60 70 80 90 100

()	

Clothing_C1 Can you commit to this action?: **Use less energy related to clothing (e.g., hang dry clothing and wash clothes in cold water) for at least one year**

	Definitely not	Absolutely yes	I already do this

0 10 20 30 40 50 60 70 80 90 100

()	

Vegetarian1_C1 Can you commit to this action?: **Eat 30% more vegetarian food (e.g., be vegetarian for one additional meal per day) for at least one year**

	Definitely not Absolutely yes I already do this<br>0 10 20 30 40 50 60 70 80 90 100
()	

ProduceRElectric_C1 Can you commit to this action?: **Produce renewable electricity (e.g., install small-scale residential solar photovoltaic)**

	Definitely not Absolutely yes I already do this<br>0 10 20 30 40 50 60 70 80 90 100
()	

CarPool_C1 Can you commit to this action?: **Car-pool/share (e.g., become a member of a car-club, reduce the number of cars in your household, or ride-share with at least 2 persons in a car) for at least one year**

	Definitely not Absolutely yes I already do this<br>0 10 20 30 40 50 60 70 80 90 100
()	

SmartMeter_C1 Can you commit to this action?: **Install smart metering (i.e., measure how much gas and electricity you're using via a remote connection to your energy supplier)**

Definitely not Absolutely yes I already do this

0 10 20 30 40 50 60 70 80 90 100

()	

ReduceWaste_C1 Can you commit to this action?: **Reduce avoidable food waste for at least one year**

	Definitely not Absolutely yes I already do this<br>0 10 20 30 40 50 60 70 80 90 100
()	

Vegetarian2_C1 Can you commit to this action?: **Eat 60% more vegetarian food (e.g., be vegetarian for two additional meals per day) for at least one year**

	Definitely not Absolutely yes I already do this<br>0 10 20 30 40 50 60 70 80 90 100
()	

LessFlight1_C1 Can you commit to this action?: **Take less transport by air (e.g., avoid medium flights, or shift from airplane to renewable train) for at least one year**

	Definitely not Absolutely yes I already do this<br>0 10 20 30 40 50 60 70 80 90 100
()	

EnergyEff_C1 Can you commit to this action?: **Increase energy efficiency (e.g., buy a more efficient car)**

	Definitely not	Absolutely yes	I already do this
	0 10 20 30 40 50 60 70 80 90 100		
()			

PublicTransport1_C1 Can you commit to this action?: **Shift from fossil fuel public transport to renewable public transport (e.g., shift from fossil fuel bus/train to renewable train)**

	Definitely not	Absolutely yes	I already do this
	0 10 20 30 40 50 60 70 80 90 100		
()			

Vegan_C1 Can you commit to this action?: **Adopt a vegan diet for at least one year**

	Definitely not	Absolutely yes	I already do this
	0 10 20 30 40 50 60 70 80 90 100		
()			

Vegetarian3_C1 Can you commit to this action?: **Adopt a vegetarian diet (e.g., go from omnivore to vegetarian) for at least one year**

	Definitely not	Absolutely yes	I already do this
	0 10 20 30 40 50 60 70 80 90 100		
()			

ElectricCar_C1 Can you commit to this action?: **Shift from fuel-powered car to a renewable electric car for at least one year**

	Definitely not Absolutely yes I already do this<br>0 10 20 30 40 50 60 70 80 90 100
()	

Meats_C1 Can you commit to this action?: **Shift to lower carbon meats (e.g., shift one third of the beef calories to either pork or poultry) for at least one year**

	Definitely not Absolutely yes I already do this<br>0 10 20 30 40 50 60 70 80 90 100
()	

PublicTransport2_C1 Can you commit to this action?: **Shift from fossil fuel car to renewable public transport (e.g., shift from fossil car to renewable train)**

	Definitely not Absolutely yes I already do this<br>0 10 20 30 40 50 60 70 80 90 100
()	

ActiveTransport_C1 Can you commit to this action?: **Shift to active transport (e.g., bike and ebike instead of taking the car) for at least one year**

Definitely not Absolutely yes I already do this

0 10 20 30 40 50 60 70 80 90 100

()	

UseRElectric_C1 Can you commit to this action?: **Use renewable electricity (e.g., buy green energy) for at least one year.**

	Definitely not	Absolutely Yes	I already do this
	0 10 20 30 40 50 60 70 80 90 100		
()			

LessFlight2_C1 Can you commit to this action?: **Take one less transatlantic flight for at least one year**

	Definitely not	Absolutely yes	I already do this
	0 10 20 30 40 50 60 70 80 90 100		
()			

Adopt_C1 Can you commit to this action?: **Not purchase/adopt a dog**

	Definitely not	Absolutely yes	I already do this
	0 10 20 30 40 50 60 70 80 90 100		
()			

Vote_C1 Can you commit to this action?: **Vote for pro-climate candidates**

	Definitely not	Absolutely yes	I already do this
	0 10 20 30 40 50 60 70 80 90 100		
()			

March_C1 Can you commit to this action?: **Attend a climate march/demonstration**

	Definitely not	Absolutely yes	I already do this
	0 10 20 30 40 50 60 70 80 90 100		
()			

Finance_C1 Can you commit to this action?: **Change your financial institution (if it invests in fossil fuels)**

	Definitely not	Absolutely yes	I already do this
	0 10 20 30 40 50 60 70 80 90 100		
()			

Donate_C1 Can you commit to this action?: **Donate to an environmental non-profit**

	Definitely not	Absolutely yes	I already do this
	0 10 20 30 40 50 60 70 80 90 100		
()			

Promote_C1 Can you commit to this action?: **Promote climate action at work**

	Definitely not	Absolutely yes	I already do this
	0 10 20 30 40 50 60 70 80 90 100		
()			

Attn1 Please set the slider bar to 100 **if you are still reading these questions.**

	Definitely not	Absolutely yes	I already do this
	0 10 20 30 40 50 60 70 80 90 100		
()			

End of Block: Commitment_PRE

Start of Block: Exp_ Ranking instructions

Q914 You will now be asked to rank each behavior on the degree to which it is effective at reducing carbon emissions compared to the other behaviors

End of Block: Exp_ Ranking instructions

Start of Block: Appliance_exp

Appliance_exp Compared to other behaviors, where would you rank the effectiveness of this action?: **Use more efficient appliances (e.g., change your lightbulbs)**

	2 3 4 5 6 7 8 9 1011121314151617181920	
	Least effective	Most effective
	1	21
()		

Page Break

Q831 Relative to other behaviors, here is the true the effectiveness of this action: **Use more efficient appliances (e.g., change your lightbulbs)**

End of Block: Appliance_exp

Start of Block: Recycle_exp

Recycle_exp Compared to other behaviors, where would you rank the effectiveness of this action?: **Comprehensively recycle for at least one year**

	Least effective	2 3 4 5 6 7 8 9 10 11 12 13 14 15 16 17 18 19 20	Most effective
		1	21
()			

Page Break

Q848 Relative to other behaviors, here is the true the effectiveness of this action: **Comprehensively recycle for at least one year**

End of Block: Recycle_exp

Start of Block: Clothing_exp

Clothing_exp Compared to other behaviors, where would you rank the effectiveness of this action?: **Use less energy related to clothing (e.g., hang dry clothing and wash clothes in cold water) for at least one year**

	Least effective	2 3 4 5 6 7 8 9 1011121314151617181920	Most effective
		1	21
()			

Page Break

Q846 Relative to other behaviors, here is the true the effectiveness of this action: **Use less energy related to clothing (e.g., hang dry clothing and wash clothes in cold water) for at least one year**

End of Block: Clothing_exp

Start of Block: Vegetarian1_exp

Vegetarian1_exp Compared to other behaviors, where would you rank the effectiveness of this action?: **Eat 30% more vegetarian food (e.g., be vegetarian for one additional meal per day) for at least one year**

	Least effective	2 3 4 5 6 7 8 9 1011121314151617181920	Most effective
		1	21
()			

Page Break

Q850 Relative to other behaviors, here is the true the effectiveness of this action: **Eat 30% more vegetarian food (e.g., be vegetarian for one additional meal per day) for at least one year**

End of Block: Vegetarian1_exp

Start of Block: ProduceRElectric_exp

ProduceRElectric_exp Compared to other behaviors, where would you rank the effectiveness of this action?: **Produce renewable electricity (e.g., install small-scale residential solar photovoltaic)**

	Least effective	2 3 4 5 6 7 8 9 1011121314151617181920	Most effective
		1 21	
()			

Page Break

Q852 Relative to other behaviors, here is the true the effectiveness of this action: **Produce renewable electricity (e.g., install small-scale residential solar photovoltaic)**

End of Block: ProduceRElectric_exp

Start of Block: CarPool_exp

CarPool_exp Compared to other behaviors, where would you rank the effectiveness of this action?: **Car-pool/share (e.g., become a member of a car-club, reduce the number of cars in your household, or ride-share with at least 2 persons in a car) for at least one year**

	Least effective	2 3 4 5 6 7 8 9 10 11 12 13 14 15 16 17 18 19 20	Most effective
		1	21
()			

Page Break

Q854 Relative to other behaviors, here is the true the effectiveness of this action: **Car-pool/share (e.g., become a member of a car-club, reduce the number of cars in your household, or ride-share with at least 2 persons in a car) for at least one year**

End of Block: CarPool_exp

Start of Block: SmartMeter_exp

SmartMeter_exp Compared to other behaviors, where would you rank the effectiveness of this action?: **Install smart metering (i.e., measure how much gas and electricity you're using via a remote connection to your energy supplier)**

	Least effective	2 3 4 5 6 7 8 9 1011121314151617181920	Most effective
		1	21
()			

Page Break

Q856 Relative to other behaviors, here is the true the effectiveness of this action: **Install smart metering (i.e., measure how much gas and electricity you're using via a remote connection to your energy supplier)**

End of Block: SmartMeter_exp

Start of Block: ReduceWaste_exp

ReduceWaste_exp Compared to other behaviors, where would you rank the effectiveness of this action?: **Reduce avoidable food waste for at least one year**

	Least effective 1 2 3 4 5 6 7 8 9 10 11 12 13 14 15 16 17 18 19 20 21 Most effective
()	

Page Break

Q858 Relative to other behaviors, here is the true the effectiveness of this action: **Reduce avoidable food waste for at least one year**

End of Block: ReduceWaste_exp

Start of Block: Vegetarian2_exp

Vegetarian2_exp Compared to other behaviors, where would you rank the effectiveness of this action?: **Eat 60% more vegetarian food (e.g., be vegetarian for two additional meals per day) for at least one year**

2 3 4 5 6 7 8 9 1011121314151617181920

Least effective | Most effective

1 21

()

Page Break

Q860 Relative to other behaviors, here is the true the effectiveness of this action: **Eat 60% more vegetarian food (e.g., be vegetarian for two additional meals per day) for at least one year**

End of Block: Vegetarian2_exp

Start of Block: LessFlight1_exp

LessFlight1_exp Compared to other behaviors, where would you rank the effectiveness of this action?: **Take less transport by air (e.g., avoid medium flights, or shift from airplane to renewable train) for at least one year**

	Least effective	2 3 4 5 6 7 8 9 1011121314151617181920	Most effective
		1	21
()			

Page Break

Q862 Relative to other behaviors, here is the true the effectiveness of this action: **Take less transport by air (e.g., avoid medium flights, or shift from airplane to renewable train) for at least one year**

End of Block: LessFlight1_exp

Start of Block: EnergyEff_exp

EnergyEff_exp Compared to other behaviors, where would you rank the effectiveness of this action?: **Increase energy efficiency (e.g., buy a more efficient car)**

	Least effective	2 3 4 5 6 7 8 9 1011121314151617181920	Most effective
		1	21
()			

Page Break

Q864 Relative to other behaviors, here is the true the effectiveness of this action: **Increase energy efficiency (e.g., buy a more efficient car)**

End of Block: EnergyEff_exp

Start of Block: PublicTransport1_exp

PublicTransport1_exp Compared to other behaviors, where would you rank the effectiveness of this action?: **Shift from fossil fuel public transport to renewable public transport (e.g., shift from fossil fuel bus/train to renewable train)**

	Least effective	2 3 4 5 6 7 8 9 1011121314151617181920	Most effective
		1	21
()			

Page Break

Q866 Relative to other behaviors, here is the true the effectiveness of this action: **Shift from fossil fuel public transport to renewable public transport (e.g., shift from fossil fuel bus/train to renewable train)**

End of Block: PublicTransport1_exp

Start of Block: Vegan_exp

Vegan_exp Compared to other behaviors, where would you rank the effectiveness of this action?: **Adopt a vegan diet for at least one year**

	Least effective	2 3 4 5 6 7 8 9 1011121314151617181920	Most effective
		1	21
()			

Page Break

Q868 Relative to other behaviors, here is the true the effectiveness of this action: **Adopt a vegan diet for at least one year**

End of Block: Vegan_exp

Start of Block: Vegetarian3_exp

Vegetarian3_exp Compared to other behaviors, where would you rank the effectiveness of this action?: **Adopt a vegetarian diet (e.g., go from omnivore to vegetarian) for at least one year**

	Least effective	2 3 4 5 6 7 8 9 1011121314151617181920	Most effective
		1	21
()			

Page Break

Q870 Relative to other behaviors, here is the true the effectiveness of this action: **Adopt a vegetarian diet (e.g., go from omnivore to vegetarian) for at least one year**

End of Block: Vegetarian3_exp

Start of Block: ElectricCar_exp

ElectricCar_exp Compared to other behaviors, where would you rank the effectiveness of this action?: **Shift from fuel-powered car to a renewable electric car for at least one year**

	Least effective	2 3 4 5 6 7 8 9 1011121314151617181920	Most effective
		1	21
()			

Page Break

Q872 Relative to other behaviors, here is the true the effectiveness of this action: **Shift from fuel-powered car to a renewable electric car for at least one year**

End of Block: ElectricCar_exp

Start of Block: Meats_exp

Meats_exp Compared to other behaviors, where would you rank the effectiveness of this action?: **Shift to lower carbon meats (e.g., shift one third of the beef calories to either pork or poultry) for at least one year**

	Least effective	2 3 4 5 6 7 8 9 1011121314151617181920	Most effective
		1	21
()			

Page Break

Q874 Relative to other behaviors, here is the true the effectiveness of this action: **Shift to lower carbon meats (e.g., shift one third of the beef calories to either pork or poultry) for at least one year**

End of Block: Meats_exp

Start of Block: PublicTransport2_exp

PublicTransport2_exp Compared to other behaviors, where would you rank the effectiveness of this action?: **Shift from fossil fuel car to renewable public transport (e.g., shift from fossil car to renewable train)**

	Least effective	2 3 4 5 6 7 8 9 1011121314151617181920	Most effective
		1	21
()			

Page Break

Q876 Relative to other behaviors, here is the true the effectiveness of this action: **Shift from fossil fuel car to renewable public transport (e.g., shift from fossil car to renewable train)**

End of Block: PublicTransport2_exp

Start of Block: ActiveTransport_exp

ActiveTransport_exp Compared to other behaviors, where would you rank the effectiveness of this action?: **Shift to active transport (e.g., bike and ebike instead of taking the car) for at least one year**

	Least effective	2 3 4 5 6 7 8 9 10 11 12 13 14 15 16 17 18 19 20	Most effective
		1	21
()			

Page Break

Q878 Relative to other behaviors, here is the true the effectiveness of this action: **Shift to active transport (e.g., bike and ebike instead of taking the car) for at least one year**

End of Block: ActiveTransport_exp

Start of Block: UseRElectric_exp

UseRElectric_exp Compared to other behaviors, where would you rank the effectiveness of this action?: **Use renewable electricity (e.g., buy green energy) for at least one year.**

	Least effective	2 3 4 5 6 7 8 9 1011121314151617181920	Most effective
		1 21	
()	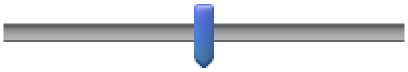		

Page Break

Q880 Relative to other behaviors, here is the true the effectiveness of this action: **Use renewable electricity (e.g., buy green energy) for at least one year.**

End of Block: UseRElectric_exp

Start of Block: LessFlight2_exp

LessFlight2_exp Compared to other behaviors, where would you rank the effectiveness of this action?: **Take one less transatlantic flight for at least one year**

	Least effective	2 3 4 5 6 7 8 9 1011121314151617181920	Most effective
		1	21
()			

Page Break

Q882 Relative to other behaviors, here is the true the effectiveness of this action: **Take one less transatlantic flight for at least one year**

End of Block: LessFlight2_exp

Start of Block: Adopt_exp

Adopt_exp Compared to other behaviors, where would you rank the effectiveness of this action?: **Not purchase/adopt a dog**

	Least effective	2 3 4 5 6 7 8 9 1011121314151617181920	Most effective
		1	21
()			

Page Break

Q884 Relative to other behaviors, here is the true the effectiveness of this action: **Not purchase/adopt a dog**

End of Block: Adopt_exp

Start of Block: Actcontrol_ Ranking Instructions

Q928 You will now be shown the rank of each behavior on the degree to which it is effective at reducing carbon emissions compared to the other behaviors

End of Block: Actcontrol_ Ranking Instructions

Start of Block: Appliance_control

Q886 Relative to other behaviors, here is the true the effectiveness of this action: **Use more efficient appliances (e.g., change your lightbulbs)**

End of Block: Appliance_control

Start of Block: Recycle_control

Q888 Relative to other behaviors, here is the true the effectiveness of this action: **Comprehensively recycle for at least one year**

End of Block: Recycle_control

Start of Block: Clothing_control

Q890 Relative to other behaviors, here is the true the effectiveness of this action: **Use less energy related to clothing (e.g., hang dry clothing and wash clothes in cold water) for at least one year**

End of Block: Clothing_control

Start of Block: Vegetarian1_control

Q892 Relative to other behaviors, here is the true the effectiveness of this action: **Eat 30% more vegetarian food (e.g., be vegetarian for one additional meal per day) for at least one year**

End of Block: Vegetarian1_control

Start of Block: ProduceRElectric_control

Q894 Relative to other behaviors, here is the true the effectiveness of this action: **Produce renewable electricity (e.g., install small-scale residential solar photovoltaic)**

End of Block: ProduceRElectric_control

Start of Block: CarPool_control

Q896 Relative to other behaviors, here is the true the effectiveness of this action: **Car-pool/share (e.g., become a member of a car-club, reduce the number of cars in your household, or ride-share with at least 2 persons in a car) for at least one year**

End of Block: CarPool_control

Start of Block: SmartMeter_control

Q898 Relative to other behaviors, here is the true the effectiveness of this action: **Install smart metering (i.e., measure how much gas and electricity you're using via a remote connection to your energy supplier)**

End of Block: SmartMeter_control

Start of Block: ReduceWaste_control

Q900 Relative to other behaviors, here is the true the effectiveness of this action: **Reduce avoidable food waste for at least one year**

End of Block: ReduceWaste_control

Start of Block: Vegetarian2_control

Q902 Relative to other behaviors, here is the true the effectiveness of this action: **Eat 60% more vegetarian food (e.g., be vegetarian for two additional meals per day) for at least one year**

End of Block: Vegetarian2_control

Start of Block: LessFlight1_control

Q904 Relative to other behaviors, here is the true the effectiveness of this action: **Take less transport by air (e.g., avoid medium flights, or shift from airplane to renewable train) for at least one year**

End of Block: LessFlight1_control

Start of Block: EnergyEff_control

Q906 Relative to other behaviors, here is the true the effectiveness of this action: **Increase energy efficiency (e.g., buy a more efficient car)**

End of Block: EnergyEff_control

Start of Block: PublicTransport1_control

Q908 Relative to other behaviors, here is the true the effectiveness of this action: **Shift from fossil fuel public transport to renewable public transport (e.g., shift from fossil fuel bus/train to renewable train)**

End of Block: PublicTransport1_control

Start of Block: Vegan_control

Q910 Relative to other behaviors, here is the true the effectiveness of this action: **Adopt a vegan diet for at least one year**

End of Block: Vegan_control

Start of Block: Vegetarian3_control

Q912 Relative to other behaviors, here is the true the effectiveness of this action: **Adopt a vegetarian diet (e.g., go from omnivore to vegetarian) for at least one year**

End of Block: Vegetarian3_control

Start of Block: ElectricCar_control

Q914 Relative to other behaviors, here is the true the effectiveness of this action: **Shift from fuel-powered car to a renewable electric car for at least one year**

End of Block: ElectricCar_control

Start of Block: Meats_control

Q916 Relative to other behaviors, here is the true the effectiveness of this action: **Shift to lower carbon meats (e.g., shift one third of the beef calories to either pork or poultry) for at least one year**

End of Block: Meats_control

Start of Block: PublicTransport2_control

Q918 Relative to other behaviors, here is the true the effectiveness of this action: **Shift from fossil fuel car to renewable public transport (e.g., shift from fossil car to renewable train)**

End of Block: PublicTransport2_control

Start of Block: ActiveTransport_control

Q920 Relative to other behaviors, here is the true the effectiveness of this action: **Shift to active transport (e.g., bike and ebike instead of taking the car) for at least one year**

End of Block: ActiveTransport_control

Start of Block: UseRElectric_control

Q922 Relative to other behaviors, here is the true the effectiveness of this action: **Use renewable electricity (e.g., buy green energy) for at least one year.**

End of Block: UseRElectric_control

Start of Block: LessFlight2_control

Q924 Relative to other behaviors, here is the true the effectiveness of this action: **Take one less transatlantic flight for at least one year**

End of Block: LessFlight2_control

Start of Block: Adopt_control

Q926 Relative to other behaviors, here is the true the effectiveness of this action: **Not purchase/adopt a dog**

End of Block: Adopt_control

Start of Block: Control_distracter

Control_page1 Please carefully read the following excerpt (you may be asked about it in the following pages).

Control_distract As soon as the great black velvet pall outside my little window was shot with grey, I got up and went downstairs; every board upon the way, and every crack in every board calling after me, "Stop thief!" and "Get up, Mrs. Joe!" In the pantry, which was far more abundantly supplied than usual, owing to the season, I was very much alarmed by a hare hanging up by the heels, whom I rather thought I caught, when my back was half turned, winking. I had no time for verification, no time for selection, no time for anything, for I had no time to spare. I stole some bread, some rind of cheese, about half a jar of mincemeat (which I tied up in my pocket-handkerchief with my last night’s slice), some brandy from a stone bottle (which I decanted into a glass bottle I had secretly used for making that intoxicating fluid, Spanish-liquorice-water, up in my room: diluting the stone bottle from a jug in the kitchen cupboard), a meat bone with very little on it, and a beautiful round compact pork pie. I was nearly going away without the pie, but I was tempted to mount upon a shelf, to look what it was

that was put away so carefully in a covered earthenware dish in a corner, and I found it was the pie, and I took it in the hope that it was not intended for early use, and would not be missed for some time.

You will be able to advance the page shortly

Control_time Timing
First Click (1)
Last Click (2)
Page Submit (3)
Click Count (4)

End of Block: Control_distracter

Start of Block: Commitment_InstructionsPOST

Q929 You will now be asked to again rate the carbon emissions-reducing behaviors on the degree to which you can commit to them.

End of Block: Commitment_InstructionsPOST

Start of Block: Commitment_POST

Appliance_C2 Can you commit to this action?: **Use more efficient appliances (e.g., change your lightbulbs)**

	Definitely not Absolutely yes I already do this
	0 10 20 30 40 50 60 70 80 90 100
()	

Recycle_C2 Can you commit to this action?: **Comprehensively recycle for at least one year**

	Definitely not Absolutely yes I already do this
	0 10 20 30 40 50 60 70 80 90 100
()	

Clothing_C2 Can you commit to this action?: **Use less energy related to clothing (e.g., hang dry clothing and wash clothes in cold water) for at least one year**

	Definitely not Absolutely yes I already do this
	0 10 20 30 40 50 60 70 80 90 100
()	

Vegetarian1_C2 Can you commit to this action?: **Eat 30% more vegetarian food (e.g., be vegetarian for one additional meal per day) for at least one year**

	Definitely not Absolutely yes I already do this
	0 10 20 30 40 50 60 70 80 90 100
()	

ProduceRElectric_C2 Can you commit to this action?: **Produce renewable electricity (e.g., install small-scale residential solar photovoltaic)**

	Definitely not Absolutely yes I already do this
	0 10 20 30 40 50 60 70 80 90 100
()	

CarPool_C2 Can you commit to this action?: **Car-pool/share (e.g., become a member of a car-club, reduce the number of cars in your household, or ride-share with at least 2 persons in a car) for at least one year**

Definitely not Absolutely yes I already do this

0 10 20 30 40 50 60 70 80 90 100

()	

SmartMeter_C2 Can you commit to this action?: **Install smart metering (i.e., measure how much gas and electricity you're using via a remote connection to your energy supplier)**

Definitely not Absolutely yes I already do this

0 10 20 30 40 50 60 70 80 90 100

()	

ReduceWaste_C2 Can you commit to this action?: **Reduce avoidable food waste for at least one year**

Definitely not Absolutely yes I already do this

0 10 20 30 40 50 60 70 80 90 100

()	

Vegetarian2_C2 Can you commit to this action?: **Eat 60% more vegetarian food (e.g., be vegetarian for two additional meals per day) for at least one year**

Definitely not Absolutely yes I already do this

	Definitely not	Absolutely yes	I already do this

0 10 20 30 40 50 60 70 80 90 100

()	

LessFlight1_C2 Can you commit to this action?: **Take less transport by air (e.g., avoid medium flights, or shift from airplane to renewable train) for at least one year**

Definitely not Absolutely yes I already do this

0 10 20 30 40 50 60 70 80 90 100

()	

EnergyEff_C2 Can you commit to this action?: **Increase energy efficiency (e.g., buy a more efficient car)**

Definitely not Absolutely yes I already do this

0 10 20 30 40 50 60 70 80 90 100

()	

PublicTransport1_C2 Can you commit to this action?: **Shift from fossil fuel public transport to renewable public transport (e.g., shift from fossil fuel bus/train to renewable train)**

Definitely not Absolutely yes I already do this

0 10 20 30 40 50 60 70 80 90 100

()	

Vegan_C2 Can you commit to this action?: **Adopt a vegan diet for at least one year**

	Definitely not					Absolutely yes					I already do this
	0	10	20	30	40	50	60	70	80	90	100
()											

Vegetarian3_C2 Can you commit to this action?: **Adopt a vegetarian diet (e.g., go from omnivore to vegetarian) for at least one year**

	Definitely not					Absolutely yes					I already do this
	0	10	20	30	40	50	60	70	80	90	100
()											

ElectricCar_C2 Can you commit to this action?: **Shift from fuel-powered car to a renewable electric car for at least one year**

	Definitely not					Absolutely yes					I already do this
	0	10	20	30	40	50	60	70	80	90	100
()											

Meats_C2 Can you commit to this action?: **Shift to lower carbon meats (e.g., shift one third of the beef calories to either pork or poultry) for at least one year**

	Definitely not					Absolutely yes					I already do this
	0	10	20	30	40	50	60	70	80	90	100
()											

PublicTransport2_C2 Can you commit to this action?: **Shift from fossil fuel car to renewable public transport (e.g., shift from fossil car to renewable train)**

	Definitely not Absolutely yes I already do this<br>0 10 20 30 40 50 60 70 80 90 100
()	

ActiveTransport_C2 Can you commit to this action?: **Shift to active transport (e.g., bike and ebike instead of taking the car) for at least one year**

	Definitely not Absolutely yes I already do this<br>0 10 20 30 40 50 60 70 80 90 100
()	

UseRElectric_C2 Can you commit to this action?: **Use renewable electricity (e.g., buy green energy) for at least one year.**

	Definitely not Absolutely Yes I already do this<br>0 10 20 30 40 50 60 70 80 90 100
()	

LessFlight2_C2 Can you commit to this action?: **Take one less transatlantic flight for at least one year**

Definitely not Absolutely yes I already do this

0 10 20 30 40 50 60 70 80 90 100

()	

Adopt_C2 Can you commit to this action?: **Not purchase/adopt a dog**

	Definitely not	Absolutely yes	I already do this
	0 10 20 30 40 50 60 70 80 90 100		
()			

Vote_C2 Can you commit to this action?: **Vote for pro-climate candidates**

	Definitely not	Absolutely yes	I already do this
	0 10 20 30 40 50 60 70 80 90 100		
()			

March_C2 Can you commit to this action?: **Attend a climate march/demonstration**

	Definitely not	Absolutely yes	I already do this
	0 10 20 30 40 50 60 70 80 90 100		
()			

Finance_C2 Can you commit to this action?: **Change your financial institution (if it invests in fossil fuels)**

Definitely not Absolutely yes I already do this

	0 10 20 30 40 50 60 70 80 90 100
()	

Donate_C2 Can you commit to this action?: **Donate to an environmental non-profit**

	Definitely not Absolutely yes I already do this<br>0 10 20 30 40 50 60 70 80 90 100
()	

Promote_C2 Can you commit to this action?: **Promote climate action at work**

	Definitely not Absolutely yes I already do this<br>0 10 20 30 40 50 60 70 80 90 100
()	

End of Block: Commitment_POST

Start of Block: Effectiveness_InstructionsPOST

Q930 You will now be asked to rate the carbon emissions-reducing behaviors on the degree to which you perceive them to be effective in terms of carbon mitigation potential.

End of Block: Effectiveness_InstructionsPOST

Start of Block: Effectiveness_POST

Appliance_E2 How effective in terms of carbon mitigation potential do you perceive this action to be?: **Use more efficient appliances (e.g., change your lightbulbs)**

	Extremely ineffective		Extremely effective		I already do this
	0 10 20 30	40	50 60	70	80 90 100
()					

Recycle_E2 How effective in terms of carbon mitigation potential do you perceive this action to be?: **Comprehensively recycle for at least one year**

	Extremely ineffective		Extremely effective		I already do this
	0 10 20 30	40	50 60	70	80 90 100
()					

Clothing_E2 How effective in terms of carbon mitigation potential do you perceive this action to be?: **Use less energy related to clothing (e.g., hang dry clothing and wash clothes in cold water) for at least one year**

	Extremely ineffective		Extremely effective		I already do this
	0 10 20 30	40	50 60	70	80 90 100
()					

Vegetarian1_E2 How effective in terms of carbon mitigation potential do you perceive this action to be?: **Eat 30% more vegetarian food (e.g., be vegetarian for one additional meal per day) for at least one year**

	Extremely ineffective	Extremely effective	I already do this
	0 10 20 30	40 50 60 70	80 90 100
()			

ProduceRElectric_E2 How effective in terms of carbon mitigation potential do you perceive this action to be?: **Produce renewable electricity (e.g., install small-scale residential solar photovoltaic)**

	Extremely ineffective	Extremely effective	I already do this
	0 10 20 30	40 50 60 70	80 90 100
()			

Page Break

CarPool_E2 How effective in terms of carbon mitigation potential do you perceive this action to be?: **Car-pool/share (e.g., become a member of a car-club, reduce the number of cars in your household, or ride-share with at least 2 persons in a car) for at least one year**

	Extremely ineffective	Extremely effective	I already do this
	0 10 20 30 40 50 60 70 80 90 100		
()			

SmartMeter_E2 How effective in terms of carbon mitigation potential do you perceive this action to be?: **Install smart metering (i.e., measure how much gas and electricity you're using via a remote connection to your energy supplier)**

	Extremely ineffective	Extremely effective	I already do this
	0 10 20 30 40 50 60 70 80 90 100		
()			

ReduceWaste_E2 How effective in terms of carbon mitigation potential do you perceive this action to be?: **Reduce avoidable food waste for at least one year**

	Extremely ineffective	Extremely effective	I already do this
	0 10 20 30 40 50 60 70 80 90 100		
()			

Vegetarian2_E2 How effective in terms of carbon mitigation potential do you perceive this action to be?: **Eat 60% more vegetarian food (e.g., be vegetarian for two additional meals per day) for at least one year**

	Extremely ineffective		Extremely effective		I already do this
	0 10 20 30	40	50 60	70 80	90 100
()					

LessFlight1_E2 How effective in terms of carbon mitigation potential do you perceive this action to be?: **Take less transport by air (e.g., avoid medium flights, or shift from airplane to renewable train) for at least one year**

	Extremely ineffective		Extremely effective		I already do this
	0 10 20 30	40	50 60	70 80	90 100
()					

EnergyEff_E2 How effective in terms of carbon mitigation potential do you perceive this action to be?: **Increase energy efficiency (e.g., buy a more efficient car)**

	Extremely ineffective		Extremely effective		I already do this
	0 10 20 30	40	50 60	70 80	90 100
()					

PublicTransport1_E2 How effective in terms of carbon mitigation potential do you perceive this action to be?: **Shift from fossil fuel public transport to renewable public transport (e.g., shift from fossil fuel bus/train to renewable train)**

	Extremely ineffective	Extremely effective	I already do this
	0 10 20 30 40 50 60 70 80 90 100		
()			

Vegan_E2 How effective in terms of carbon mitigation potential do you perceive this action to be?: **Adopt a vegan diet for at least one year**

	Extremely ineffective	Extremely effective	I already do this
	0 10 20 30 40 50 60 70 80 90 100		
()			

Vegetarian3_E2 How effective in terms of carbon mitigation potential do you perceive this action to be?: **Adopt a vegetarian diet (e.g., go from omnivore to vegetarian) for at least one year**

	Extremely ineffective	Extremely effective	I already do this
	0 10 20 30 40 50 60 70 80 90 100		
()			

ElectricCar_E2 How effective in terms of carbon mitigation potential do you perceive this action to be?: **Shift from fuel-powered car to a renewable electric car for at least one year**

Extremely ineffective | Extremely effective | I already do this

0 10 20 30 40 50 60 70 80 90 100

()

Meats_E2 How effective in terms of carbon mitigation potential do you perceive this action to be?: **Shift to lower carbon meats (e.g., shift one third of the beef calories to either pork or poultry) for at least one year**

	Extremely ineffective	Extremely effective	I already do this

0 10 20 30 40 50 60 70 80 90 100

()

PublicTransport2_E2 How effective in terms of carbon mitigation potential do you perceive this action to be?: **Shift from fossil fuel car to renewable public transport (e.g., shift from fossil car to renewable train)**

	Extremely ineffective	Extremely effective	I already do this

0 10 20 30 40 50 60 70 80 90 100

()

ActiveTransport_E2 How effective in terms of carbon mitigation potential do you perceive this action to be?: **Shift to active transport (e.g., bike and ebike instead of taking the car) for at least one year**

	Extremely ineffective	Extremely effective	I already do this

0 10 20 30 40 50 60 70 80 90 100

()	

UseRElectric_E2 How effective in terms of carbon mitigation potential do you perceive this action to be?: **Use renewable electricity (e.g., buy green energy) for at least one year.**

	Extremely ineffective				Extremely effective				I already do this	
	0	10	20	30	40	50	60	70	80	90	100
()											

LessFlight2_E2 How effective in terms of carbon mitigation potential do you perceive this action to be?: **Take one less transatlantic flight for at least one year**

	Extremely ineffective				Extremely effective				I already do this	
	0	10	20	30	40	50	60	70	80	90	100
()											

Adopt_E2 How effective in terms of carbon mitigation potential do you perceive this action to be?: **Not purchase/adopt a dog**

	Extremely ineffective				Extremely effective				I already do this	
	0	10	20	30	40	50	60	70	80	90	100
()											

Vote_E2 How effective in terms of carbon mitigation potential do you perceive this action to be?: **Vote for pro-climate candidates**

	Extremely ineffective	Extremely effective	I already do this
	0 10 20 30 40 50 60 70 80 90 100		
()			

March_E2 How effective in terms of carbon mitigation potential do you perceive this action to be?: **Attend a climate march/demonstration**

	Extremely ineffective	Extremely effective	I already do this
	0 10 20 30 40 50 60 70 80 90 100		
()			

Finance_E2 How effective in terms of carbon mitigation potential do you perceive this action to be?: **Change your financial institution (if it invests in fossil fuels)**

	Extremely ineffective	Extremely effective	I already do this
	0 10 20 30 40 50 60 70 80 90 100		
()			

Donate_E2 How effective in terms of carbon mitigation potential do you perceive this action to be?: **Donate to an environmental non-profit**

	Extremely ineffective	Extremely effective	I already do this

	0	10	20	30	40	50	60	70	80	90	100
()											

Promote_E2 How effective in terms of carbon mitigation potential do you perceive this action to be?: **Promote climate action at work**

	Extremely ineffective	Extremely effective	I already do this

	0	10	20	30	40	50	60	70	80	90	100
()											

End of Block: Effectiveness_POST

Start of Block: Plasticity_InstructionsPOST

Q931 You will now be asked to rate the carbon emissions-reducing behaviors on how easy or difficult it would be for you to do this action.

End of Block: Plasticity_InstructionsPOST

Start of Block: Plasticity_POST

Appliance_P How difficult or easy would it be for you to do this action?: **Use more efficient appliances (e.g., change your lightbulbs)**

	Extremely difficult	Extremely easy	I already do this

	0	10	20	30	40	50	60	70	80	90	100
()											

Recycle_P How difficult or easy would it be for you to do this action?: **Comprehensively recycle for at least one year**

	Extremely difficult	Extremely easy	I already do this
	0 10 20 30 40 50 60 70 80 90 100		
()			

Clothing_P How difficult or easy would it be for you to do this action?: **Use less energy related to clothing (e.g., hang dry clothing and wash clothes in cold water) for at least one year**

	Extremely difficult	Extremely easy	I already do this
	0 10 20 30 40 50 60 70 80 90 100		
()			

Vegetarian1_P How difficult or easy would it be for you to do this action?: **Eat 30% more vegetarian food (e.g., be vegetarian for one additional meal per day) for at least one year**

	Extremely difficult	Extremely easy	I already do this
	0 10 20 30 40 50 60 70 80 90 100		
()			

ProduceRElectric_P How difficult or easy would it be for you to do this action?: **Produce renewable electricity (e.g., install small-scale residential solar photovoltaic)**

	Extremely difficult	Extremely easy	I already do this
	0 10 20 30 40 50 60 70 80 90 100		

()	

CarPool_P How difficult or easy would it be for you to do this action?: **Car-pool/share (e.g., become a member of a car-club, reduce the number of cars in your household, or ride-share with at least 2 persons in a car) for at least one year**

	Extremely difficult	Extremely easy	I already do this
	0 10 20 30 40 50 60 70 80 90 100		
()			

SmartMeter_P How difficult or easy would it be for you to do this action?: **Install smart metering (i.e., measure how much gas and electricity you're using via a remote connection to your energy supplier)**

	Extremely difficult	Extremely easy	I already do this
	0 10 20 30 40 50 60 70 80 90 100		
()			

ReduceWaste_P How difficult or easy would it be for you to do this action?: **Reduce avoidable food waste for at least one year**

	Extremely difficult	Extremely easy	I already do this
	0 10 20 30 40 50 60 70 80 90 100		

()	

Vegetarian2_P How difficult or easy would it be for you to do this action?: **Eat 60% more vegetarian food (e.g., be vegetarian for two additional meals per day) for at least one year**

	Extremely difficult				Extremely easy				I already do this		
	0	10	20	30	40	50	60	70	80	90	100
()											

LessFlight1_P How difficult or easy would it be for you to do this action?: **Take less transport by air (e.g., avoid medium flights, or shift from airplane to renewable train) for at least one year**

	Extremely difficult				Extremely easy				I already do this		
	0	10	20	30	40	50	60	70	80	90	100
()											

EnergyEff_P How difficult or easy would it be for you to do this action?: **Increase energy efficiency (e.g., buy a more efficient car)**

	Extremely difficult				Extremely easy				I already do this		
	0	10	20	30	40	50	60	70	80	90	100
()											

PublicTransport1_P How difficult or easy would it be for you to do this action?: **Shift from fossil fuel public transport to renewable public transport (e.g., shift from fossil fuel bus/train to renewable train)**

	Extremely difficult	Extremely easy	I already do this
	0 10 20 30 40 50 60 70 80 90 100		
()			

Vegan_P How difficult or easy would it be for you to do this action?: **Adopt a vegan diet for at least one year**

	Extremely difficult	Extremely easy	I already do this
	0 10 20 30 40 50 60 70 80 90 100		
()			

Vegetarian3_P How difficult or easy would it be for you to do this action?: **Adopt a vegetarian diet (e.g., go from omnivore to vegetarian) for at least one year**

	Extremely difficult	Extremely easy	I already do this
	0 10 20 30 40 50 60 70 80 90 100		
()			

ElectricCar_P How difficult or easy would it be for you to do this action?: **Shift from fuel-powered car to a renewable electric car for at least one year**

	Extremely difficult	Extremely easy	I already do this
	0 10 20 30 40 50 60 70 80 90 100		
()			

Meats_P How difficult or easy would it be for you to do this action?: **Shift to lower carbon meats (e.g., shift one third of the beef calories to either pork or poultry) for at least one year**

	Extremely difficult	Extremely easy	I already do this
	0 10 20 30 40 50 60 70 80 90 100		
()			

PublicTransport2_P How difficult or easy would it be for you to do this action?: **Shift from fossil fuel car to renewable public transport (e.g., shift from fossil car to renewable train)**

	Extremely difficult	Extremely easy	I already do this
	0 10 20 30 40 50 60 70 80 90 100		
()			

ActiveTransport_P How difficult or easy would it be for you to do this action?: **Shift to active transport (e.g., bike and ebike instead of taking the car) for at least one year**

Extremely difficult   Extremely easy   I already do this

	0	10	20	30	40	50	60	70	80	90	100
()											

UseRElectric_P How difficult or easy would it be for you to do this action?: **Use renewable electricity (e.g., buy green energy) for at least one year.**

Extremely difficult | Extremely easy | I already do this

	0	10	20	30	40	50	60	70	80	90	100
()											

LessFlight2_P How difficult or easy would it be for you to do this action?: **Take one less transatlantic flight for at least one year**

Extremely difficult | Extremely easy | I already do this

	0	10	20	30	40	50	60	70	80	90	100
()											

Adopt_P How difficult or easy would it be for you to do this action?: **Not purchase/adopt a dog**

Extremely difficult | Extremely easy | I already do this

	0	10	20	30	40	50	60	70	80	90	100
()											

Vote_P How difficult or easy would it be for you to do this action?: **Vote for pro-climate candidates**

	Extremely difficult	Extremely easy	I already do this
	0 10 20 30 40 50 60 70 80 90 100		
()			

March_P How difficult or easy would it be for you to do this action?: **Attend a climate march/demonstration**

	Extremely difficult	Extremely easy	I already do this
	0 10 20 30 40 50 60 70 80 90 100		
()			

Finance_P How difficult or easy would it be for you to do this action?: **Change your financial institution (if it invests in fossil fuels)**

	Extremely difficult	Extremely easy	I already do this
	0 10 20 30 40 50 60 70 80 90 100		
()			

Donate_P How difficult or easy would it be for you to do this action?: **Donate to an environmental non-profit**

	Extremely difficult	Extremely easy	I already do this
	0 10 20 30 40 50 60 70 80 90 100		
()			

Promote_P How difficult or easy would it be for you to do this action?: **Promote climate action at work**

	Extremely difficult	Extremely easy	I already do this
	0 10 20 30 40 50 60 70 80 90 100		
()			

End of Block: Plasticity_POST

Start of Block: Demographics

Demo_instruct The following section includes some questions about your background and demographics. These questions may not seem particularly relevant to the tasks that you completed today. However, knowing the demographics of the people who take part in our research helps us understand who our participant sample represents. This is important in understanding the extent to which our findings might be specific to certain groups of people (e.g., undergraduate students), or whether they might generalize to wider populations.

Gender What is your gender?

- Male (1)
- Female (2)
- Prefer not to say (3)
- Non-binary/third gender/other (4) ____

Age How old are you? (please enter a number)

____

Edu How many years of formal education have you completed?

- 0-6 (up to grade school/elementary school) (1)
- 7-12 (up to high school) (2)
- 13-16 (college/undergraduate university/certificate training) (3)
- More than 17 years (doctorate degree, medical degree, etc.) (4)
- Prefer not to answer (5)

Politics What political party do you identify with?

- ○ Democratic (1)
- ○ Republican (2)
- ○ Other (3)

Page Break

Ideology What is your political orientation for the issues listed below? Please note, by "liberal" we mean classically left-wing, and by "conservative", we mean classically right-wing.

	Extremely liberal/left-wing	Moderate	Extremely conservative/right-wing	Prefer not to respond
	0 10 20 30 40 50 60 70 80 90 100			
For social issues (e.g., health care, education, etc.) (1)				
For economic issues (e.g., taxes) (9)				

Q72 We are also interested in learning about you/your family. Please answer the following questions to the best of your abilities:

Income
What is your total yearly family/household income?

- Less than $10,000 (1)
- $10,000 to $14,999 (2)
- $15,000 to $24,999 (3)
- $25,000 to $49,999 (4)
- $50,000 to $99,999 (5)
- $100,000 to $149,999 (6)
- $150,000 to $199,999 (7)
- $200,000 or more (8)
- Prefer not to respond (9)

Indirect_SES Do you own/have access to these items **in your home**? (check all that apply)

- Separate room for kitchen (1)
- Washing machine (2)
- Vacuum cleaner (3)
- Freezer/deep freeze (4)
- Personal computer (5)
- Bathroom (6)
- Television (7)

MacArthur_instruct **Instructions:** Think of this ladder as representing where people stand in the United States. At the **top** of the ladder are the people who are the best off – those who have the most money, the most education, and the most respected jobs. At the **bottom** are the people who are the worst off – those who have the least money, least education, the least respected jobs, or no job. The higher up you are on this ladder, the closer you are to the people at the very top; the lower you are, the closer you are to the people at the very bottom.

MacArthur_SES
Where would you place yourself on this ladder? Please choose the rung where you think you stand at this time in your life relative to other people in the United States.

- Rung 10 (Top) People here are the best off (10)
- Rung 9 (9)
- Rung 8 (8)
- Rung 7 (7)
- Rung 6 (6)
- Rung 5 (5)
- Rung 4 (4)
- Rung 3 (3)
- Rung 2 (2)
- Rung 1 (Bottom) People here are the worst off (1)

Page Break

CC_Threat To what degree do you think climate change poses a threat to human civilization?

	Not at all										Extremely
	0	10	20	30	40	50	60	70	80	90	100
(1)											

Page Break

PerceivedConsensus To the best of your knowledge, what percentage of climate scientists have concluded that human-caused climate change is happening?

	0	10	20	30	40	50	60	70	80	90	100
Percentage (1)											

End of Block: Demographics
